# Supplementary material for: Graph pangenome captures missing heritability and empowers tomato breeding
Source: Nature. 2022 Jun 8;606(7914):527–34. doi: 10.1038/s41586-022-04808-9 (PMC9200638; doi:10.1038/s41586-022-04808-9)
Supplement: Supplementary file 1 — Supplementary Methods and Results are provided within Supplementary Notes 1–9 and Supplementary Figs. 1–20. [file 41586_2022_4808_MOESM1_ESM.pdf]

nature portfolio

https://doi.org/10.1038/s41586-022-04808-9

Supplementary information

Graph pangenome captures missing heritability and empowers tomato breeding

In the format provided by the authors and unedited

Supplementary Notes for

Graph pangenome captures missing heritability and empowers tomato breeding

Yao Zhou[1,*], Zhiyang Zhang[1,*], Zhigui Bao[1,*], Hongbo Li[1], Yaqing Lyu[1], Yanjun Zan[1,9], Yaoyao Wu[1], Lin Cheng[1], Yuhan Fang[1], Kun Wu[1], Jinzhe Zhang[2], Hongjun Lyu[1,12], Tao Lin[3], Qiang Gao[4], Surya Saha[5], Lukas Mueller[5], Zhangjun Fei[5,6], Thomas Städler[8], Shizhong Xu[11], Zhiwu Zhang[7], Doug Speed[10], Sanwen Huang[1,#]

Affiliations:

[1]Shenzhen Branch, Guangdong Laboratory of Lingnan Modern Agriculture, Genome Analysis Laboratory of the Ministry of Agriculture and Rural Affairs, Agricultural Genomics Institute at Shenzhen, Chinese Academy of Agricultural Sciences, Shenzhen, Guangdong 518120, China

[2]Institute of Vegetables and Flowers, Chinese Academy of Agricultural Sciences, Key Laboratory of Biology and Genetic Improvement of Horticultural Crops of the Ministry of Agriculture, Sino-Dutch Joint Laboratory of Horticultural Genomics, Beijing 100081, China

[3]State Key Laboratory of Agrobiotechnology, College of Horticulture, China Agricultural University, Beijing 100081, China

[4]Boke Biotech Co., Wuxi, Jiangsu 214000, China

[5]Boyce Thompson Institute, Cornell University, Ithaca, NY 14853, USA

[6]U.S. Department of Agriculture-Agricultural Research Service, Robert W. Holley Center for Agriculture and Health, Ithaca, NY 14853, USA

[7]Department of Crop and Soil Sciences, Washington State University, Pullman, WA, USA

[8]Institute of Integrative Biology & Zurich–Basel Plant Science Center, ETH Zurich, 8092, Zurich Switzerland

[9]Umeå Plant Science Center, Department of Forestry Genetics and Plant Physiology, Swedish University of Agricultural Sciences, Umeå 90736, Sweden

[10]Quantitative Genetics and Genomics (QGG), Aarhus University, Aarhus Denmark

[11]Department of Botany and Plant Sciences, University of California, Riverside, CA 92521, USA

[12]Institute of Vegetables, Shandong Academy of Agricultural Sciences, Shandong Province Key Laboratory for Biology of Greenhouse Vegetables, Shandong Branch of National Improvement Center for Vegetables, Huang-Huai-Hai Region Scientific Observation and Experimental Station of Vegetables, Ministry of Agriculture and Rural Affairs, Jinan, Shandong, 250100, China

[#]Corresponding author: huangsanwen@caas.cn (S.H.)

[*]These authors contributed equally to this work.

Contents

Supplementary note 1: Material and methods 4
1.1 Plant materials 4
1.2 DNA preparation and sequencing 4
1.3 Assembly pipeline using HiFi reads 4
1.4 Scaffolding 5
1.5 Genome assemblies 5
1.6 Transposable element (TE) annotation 5
1.7 Protein-coding gene annotation 6
1.8 Functional annotation of gene models 8
1.9 Gene family analysis 8
Supplementary note 2: Genome characteristics of SL5.0 9
2.1 Genome assembly and evaluation 9
2.2 Repeat elements annotation 9
2.3 Protein-coding gene annotation 10
Supplementary note 3: Pan-genome analysis of all assemblies 11
3.1 RNA mapping rates 11
3.2 K-mer spectrum analysis 11
3.3 LTR Assembly Index 11
3.4 Collinearity analysis 11
3.5 BUSCO evaluation 12
3.6 Gene family analysis 12
Supplementary note 4: Construction of tomato graph pangenome (TGG) 13
4.1 SNPs and InDels calling from HiFi reads 13
4.2 SVs 13
4.3 Construction of TGG1.0 13
4.4 Variant calling with short reads 14
4.5 Construction of TGG1.1 14
Supplementary note 5: Benchmark study of variant calling 15
5.1 Simulation of short reads 15
5.2 Evaluation 15
5.3 Comparison of SNPs and InDels from SL5.0 and TGG1.1 15
Supplementary note 6: Heritability analysis 17
6.1 Phenotype simulation 17
6.2 Effect of the parameters 17
6.3 Effect of incomplete LD 17
6.4 The effect of power 18
6.5 Heritability of *cis* regions 18
6.5 Heritability of leading variants 18
6.5 Heritability of local variants 18
Supplementary note 7: Co-expression network analysis 19

Supplementary note 8: a web-based tomato database 20
Supplementary note 9: DNA capture array of SVs 21
9.1 Marker selection for SVs array panel 21
9.2 Conceptual design of probes for tomato SV gene panel 21
Supplementary figures 22

Supplementary note 1: Material and methods

1.1 Plant materials

To exhaustively and precisely identify tomato genetic variants, we collected 32 diverse tomato accessions, including the accession Heinz 1706 used as reference genome. A total of 24 accessions (9 BIG, 8 CER, and 7 PIM) were selected according to the phylogeny tree constructed for the 706 accessions with short-reads sequencing data (Supplementary Fig. 1). Moreover, a total of 7 accessions (6 BIG and 1 PIM) without short reads were selected because they are fundamental materials used in our laboratory. Seeds of the 32 accessions were sowed in a greenhouse at the Agricultural Genomics Institute at Shenzhen, Chinese Academy of Agricultural Sciences (CAAS), in plastic flats, and placed in a dark environment until the seeds germinated. The seedlings were transferred to a greenhouse under long-day conditions (16-h light/8-h dark) and a controlled temperature (20-28 °C).

1.2 DNA preparation and sequencing

To construct HiFi SMRTbell libraries for generating high-accuracy long reads, fresh leaves of the 32 accessions were collected, flash frozen in liquid nirogen and stored at -80 °C in an ultra-low-temperature freezer. The SMRTbell libraries were prepared using SMRTbell Template PrepKit 1.0-SPv3 (PacBio kit ID: 100-991-900) following the manufacturer's instruction and sequenced on the PacBio Sequel II platform (Pacific Biosciences, Menlo Park, CA, USA). We generated the HiFi reads with PacBio official software ccs (https://github.com/PacificBiosciences/ccs). The yield of the 32 accessions ranged from 20 Gb to 39 Gb, corresponding to approximately 25 to 49x of the genome (Table S2).

1.3 Assembly pipeline using HiFi reads

We developed a custom pipeline for genome assembly using HiFi reads (Supplementary Fig. 3). Preliminary assemblies from HiFi reads were generated utilising three different assembly algorithms, including Hifiasm (v0.13)[1], HiCanu (v2.0)[2] and Flye (v2.7)[3]. The Gap-free long-read assembler (GALA) pipeline[4] was used to correct for potential mis-assemblies. Given that a total of 9 accessions had high genotype heterozygosity (larger than 2%), to avoid potential errors of assemblies, we used purge_dups (v1.2.3)[5], and only the primary haplotype was

retained for each accession. Finally, all assemblies were mapped to the NT database and potential pollution sequences were removed.

1.4 Scaffolding

For the reference genome (that of Heinz 1706), raw Hi-C reads were trimmed and filtered by fastp[6], with the default parameters. The clean reads were subsequently aligned to contigs using the Juice pipeline[7] (enzyme site: Dpn II). The contigs were ordered and oriented to 12 pseudomolecules by the 3d-DNA pipeline (v180922)[8], when visualizing the corresponding Hi-C contact matrix by the use of Juicebox (v1.11.9)[7], we did not find any potential mis-assembly within contigs, reflecting the robust assembly of our pipeline. For the remaining 31 accessions without Hi-C data, the contigs were ordered and orientated to chromosomes using the reference-guided software Ragtag[9]. Briefly, the accessions belonging to BIG or CER groups were directly guided using the Heinz 1706 assembly, while the remaining PIM groups were guided using LA2093 assembly[10].

1.5 Genome assemblies

The data from previously published genome assemblies comprising 14 accessions[11] assembled using Oxford Nanopore Technology (ONT) reads and one PIM (LA2093) assembled using Continuous Long Read (CLR) reads were added to our analysis. Since accession M82 was also included in our high-quality HiFi sequencing (contig N50: 33.71 Mb), we excluded the M82 genome assemblied using ONT reads (contig N50: 1.70 Mb). A total of 46 *de novo* assemblies were ultimately used in subsequent analysis (24 BIG, 11 CER, and 11 PIM accessions) (Table S3).

1.6 Transposable element (TE) annotation

To annotate the TEs for each assembly, the Extensive de-novo TE Annotator (EDTA v1.9.4) pipeline[12] was used, in which TEs identified from structure-based or homology-based approaches were integrated (Supplementary Fig. 4). Potential intact long terminal repeat (LTR) retrotransposons (LTR-RT) were distinguished by LTRharvest_parallel (v1.5.10)[13] and LTR_FINDER_parallel (v20190525)[14], and potential false candidate LTRs were filtered and removed with LTR_retriever (v2.9.0)[15]. Terminal-inverted repeat (TIR) elements were

annotated by TIR-Learner (v2.5)[16] implemented in EDTA (with the parameter: --species others). Helitron elements were identified by HelitronScanner (v1.1)[17], and the remaining TE sequences were identified by RepeatModeler (v2.0.1)[18]. In addition, RepeatMasker (v4.1.0, -e ncbi -pa 64 -q -no_is -div 40)[19] was applied to perform homolog annotation with the EDTA TE library (EDTA.TElib.fa)[12]. Finally, the homology-based annotations from RepeatMasker[19] and structure-based annotations from EDTA were combined to form a comprehensive TE annotations for each accession.

1.7 Protein-coding gene annotation

1.7.1 Homology-based prediction

A total of 83 RNA-seq dataset[20,21] were collected covering various tissues, including flowers, leaves, stems, roots, fruits, pollen and seeds, from *Solanum lycopersicum*, *Solanum lycopersicum* var. *cerasiforme*, and *Sonalum pimpinellifolium* (Table S14). RNA sequences were aligned to the softmasked assembly using Hisat2 (v2.10.2)[22] and subsequently assembled into transcripts by StringTie (v1.3.0)[23]. TACO[24] was applied to merge stringtie gtf files (--filter-splice-juncs) of all the dataset. The sequences of the proteins from SwissProt Viridiplantae database, three *Solnaum* species (*Solanum lycopersicum* cv. Heniz 1706 ITAG4.0 (ref. [25]), *Solanum pimpinellifolium* cv. LA2093 (ref. [10]), *Solanum tuberosum* DM v6.1 (ref. [26]), and *Arabidopsis thaliana* (Araport11_genes.201606) were integrated, and a non-redundant protein set with 143,276 protein sequences were generaged by using the CD-HIT[27] (-c 0.99). Furthermore, 300,764 Expressed Sequence Tags (ESTs) retrieved from the NCBI (date: 2020-12-22) were set as EST evidence in the MAKER[28] pipeline.

1.7.2 Ab initio prediction

BRAKER (v2.1.4)[29] was used with the above homology-based alignments as input data, and predictions were generated in GFF3 format (--gff3). Repeat masking and SNAP training were executed in the MAKER pipeline[28]. ESTs were used as support evidence, while TACO[24] were used to merged gtf and transform them into gff3 by gffread[23]. All the protein sequences were set as protein-supporting evidence. The p_score_limit and en_score_limit parameters in maker_bopts.ctl were set to 50. In repeatmasking process, the annotated TEs sequences were

hardmasked and the remaining simple repeat sequences were reserved by MAKER in softmasking (model_org=simple). By utilising all RNA-seq and proteins hints, genes structure (est2genome=1, protein2genome=1) were annotated. Gene models whose AED value smaller than 0.1 were extracted and used as SNAP training set.

1.7.3 Integration homology-based and *ab initio* prediction

To obtain high-quality annotation of protein-coding genes, the second round of MAKER pipeline was carried out with gff3 data from the first round of MAKER. Ab initio gene models were constructed from the results of the second round of MAKER, including the predictions of BRAKER and GeneMark-ET[30] (pred_gff=braker.gff, genemark-et.gff3). SNAP traing models from the first round and Augutus models from the BRAKER pipeline were used. The integrated gene models were further filtered based their AED values. The predicted genes with AED values less than 0.5 were retained.

1.7.4 Pooled RNA-seq and assembly model (PRAM) annotation

To discover novel transcripts located in the intergenic region of SL5.0, PRAM is applied which based on on a single step that encompasses all transcript construction[31]. A total of 332 RNA-seq sequences (217 BIG, 98 CER, and 17 PIM accessions, Table S15)[32], were filtered by fastp[6] and the remaining clean reads were aligned to SL5.0 with STAR[33]. The alignment files were used as input files for PRAM according to the pipeline as follows: 1) intergenic genomic ranges were defined; 2) BAM files with reads alignment in intergenic regions were extracted; 3) novel transcript models were predicting from the extracted BAM files; 4) predicted transcript models that had more than two exons and that were longer than 200 bp were retained. The expression levels of novel predicted genes were ultimately calculated with Stringtie[23]. Transcripts were discarded if they did not express across all 332 accessions (TPM = 0). TEsorter[34] was used to remove potential TEs, and repeat elements that could be aligned to the repeat database were filtered and removed. To predict the gene structure of transcripts and filter those without coding ability, Transdecoder software[35] was used as follows: 1) ORF with more than 100 amino acids were retained; 2) Homologous to the plant uniport sequences were retained; 3) ORFs didn't have a minus-strand ORF on a spliced transcript structure were retained. Finally, a total of 298

novel transcripts were predicted and integrated into previous annotation of SL5.0, which is designated as ITAG5.0.

1.8 Functional annotation of gene models

The gene function was assigned according to the best match by aligning protein sequences to UniProtKB/SwissProt database[36] using DIAMOND[37] (e-value ≤ 1e−5). The motifs and domains of predicted genes were annotated using InterProScan (v5.36)[38]. Finally, the Gene Ontology (GO) IDs for each gene were assigned according to the corresponding InterPro entry.

1.9 Gene family analysis

The core and dispensable gene sets among the 46 accessions were generated by the software Orthodfinder[39]. Only the longest isofrom of each gene was used for each accession. The cd-hit-est function of CD-HIT (v4.6) toolkit[27] (–c 1 –aS 1) was used to remove redundent sequences with 100% similarity. Then, protein sequences of the retained genes were subjected to homologous searching by DIAMOND[37] (-evalue 1e-5). Orthodfinder (-I 1.5) was used to deal with the DIAMOND result to infer gene family clustering. Gene families sharing in all accessions were defined as core gene families; Families presenting over 90% accessions (42~45 accessions) were defined as softcore gene families; Families missing in more than 2 accessions were defined as dispensable gene families (2~41), and those that only existed in one accession were defined as private gene families including the unassgined gene.

Supplementary note 2: Genome characteristics of SL5.0

2.1 Genome assembly and evaluation

For the reference accession Heinz 1706, approximately 43-fold more genome size HiFi reads were generated from one cell on the PacBio sequel II platform. Hifiasm[1], HiCanu[2], and Flye[3] ultimately assembled a total of 3,730, 8,172, and 300 contigs, respectively, with raw assembly size of 926.19 Mb (N50: 39,099,916 bp), 980.39 Mb (N50: 19,861,914 bp), and 787.16 Mb (N50: 11,066,058 bp), respectively. The three assemblies were then integrated by GALA[4] into a highly continuous assembly and further corrected by chromosome conformation capture (Hi-C) technologies, and the contigs were oriented to 12 pseudomolecules with 800.12 Mb size. Unanchored contigs were aligned to the 12 chromosomes, and repeat sequences were removed. The remaining unanchored contigs (1.70 Mb in size) were attached to Chr0 with a 100-bp gap. To validate the completeness of the SL5.0 assembly, the k-mer occurrence of short reads on assembly was analysed for both SL4.0 and SL5.0. The k-mer spectrums of both SL4.0 and SL5.0 exhibited a homozygous peak at 37x, while the SL5.0 had higher integrity (99.83%) than the SL4.0 (98.71%) did (Supplementary Fig. 5). We further assessed the completeness of BAC clones. The sequences for a total of 2,764 high-throughput genomic sequence (HTGS) phase 3 BACs were downloaded (ftp://ftp.solgenomics.net/genomes/Solanum_lycopersicum/Heinz1706/bacs/). The completeness of the BACs for the assembly of SL4.0 and SL5.0 was evaluated by software bacValidataion (https://github.com/skoren/bacValidation). SL5.0 resolved more BACs (88.02%) than SL4.0 (85.78%) did (Table S1). SL5.0 also had better continuity than SL4.0, with merely 31 gaps on the 12 chromosomes. To further evaluate the assembly quality (QV) of SL4.0 and SL5.0, an reference-free k-mer method (Merqury[40]) was applied using the short reads of Heinz 1706 (SRR404081).

2.2 Repeat elements annotation

The EDTA pipeline[12] annotated approximately 491.27 Mb (61.27%) of the SL5.0 as repetitive elements, while 488.76 Mb (62.46%) were annotated in the same manner in SL4.0. LTR retrotransposons elements constituted the most repeat elements (30.27%) in SL5.0. In addition,

SL5.0 had fewer unknown repeats than SL4.0 did (4.45% vs 5.51%), indicating that the former was of better quality and had better classification (Table S4). There was 15 Mb worth of more sequences uniquely assembled by SL5.0, of which 93% were repetitive sequences. These repeat sequences were widely distributed across the genome.

2.3 Protein-coding gene annotation

There are 34,727 protein-coding genes in ITAG2.3, which were annotated by the international Tomato Annotation Group (ITAG) with manual inspection. This annotation was updated when improvements to the genome assembly were made. The most recent version ITAG4.0 (ref. [25]) contains 34,075 protein-coding genes. We annotated SL5.0 following the pipeline of ITAG4.0. As described above, publicly available expression profiles from a wide range of tomato tissues, including roots, leaves, flower, and callus were used to generate a comprehensive and complete annotation. Given that the Solyc IDs of ITAG2.4 have been extensively used in tomato research community, we reserved the ITAG2.4 gene IDs by mapping ITAG2.4 gene models to the SL5.0 reference genome. A total of 29,085 genes out of 34,725 genes from chromosomes of ITAG2.4 ultimately migrated, and the remaining 7,563 genes in SL5.0 were named in the same way as those in ITAG2.4. There were 7,911 novel genes in ITAG5.0, along with 28,737 genes reserved from ITAG4.0.

The BUSCO[41] and RNA-seq mapping rates were used to assess the completeness of the assembly and the annotation of SL5.0. We found that both BUSCO and the mapping rates marginal increased in SL5.0 compared with SL4.0, which is consistence with the results of k-mer analysis.

Supplementary note 3: Pan-genome analysis of all assemblies

3.1 RNA mapping rates

To assess the assembly quality of each accession, we determined the RNA-seq mapping rates. The sequences for a total of 83 public Sequence Read Archive (SRA) paired-end samples of tomato were downloaded from NCBI and aligned to all the 46 assemblies using Hisat2 (ref. [22]). The mapping rates ranged from 95.0% to 99.8% (Supplementary Fig. 6). These high mapping rates indicated the complexity of protein coding genes in our assemblies.

3.2 K-mer spectrum analysis

The reference-free quality control (QC) toolkit Kmer Analysis Tools (KAT)[42] was applied to generate 31-mer spectra plots using HiFi reads. The k-mer completeness of 32 *de novo* assemblies ranged from 99.10% to 99.76% (Table. S2). All the 32 accessions showed only one single-copy main peak (Supplementary Fig. 7). These results from sequencing reads provided evidence supporting the high quality of genome assembly.

3.3 LTR Assembly Index

We annotated the repeat sequences for all assemblies (Supplementary Fig. 8). To estimate the contiguity of repetitive elements across assemblies, an LTR assembly index (LAI) was calculated for all 46 assemblies as described in a previous study[10,11,25]. We found that the LAI statistics of the 32 assemblies using HiFi reads (average LAI: 13.93) were better than those of the 15 assemblies using ONT/CLR reads (average LAI: 12.44) (Supplementary Fig. 9), representing the high quality of assemblies using HiFi reads.

3.4 Collinearity analysis

As the assemblies of SL5.0 and LA2093 were supported by Hi-C data, we aligned the 34 assemlies from BIG and CER group to SL5.0 with minimap2 (ref. [43]) (-x asm5), while the 10 assemblies from PIM were mapped to LA2093. The alignment files were further filtered to eliminate small fragements with reference lengths less than 10 kb or query lengths less than 400 kb. To visulized the collinearity results, we generated a dot-plot of each pairwise comprison using the software dotPlotly (https://github.com/tpoorten/dotPlotly) (Supplementary Fig. 10). We observed that the assemblies from the same group have the silimar patterns, regardless of

which platform was used for sequencing, reflecting the effectiveness of reference-based odering and oritention.

3.5 BUSCO evaluation

To evaluate the protein-coding gene annotations, BUSCO[41] (v4.1.4, -l solanaceae_odb10 -m genome -c 64) analysis was performed for all 46 assemblies (Supplementary Fig. 11). There was only a slight difference among assemblies, with the complete BUSCO capture ranging from 97.10%~97.70% (Table S5). These results indicated that the long-read sequencing techonogy performed well for these conserved genes, indicating that the improvement in assemblies from HiFi reads was achieved mainly by resolving the complex regions.

3.6 Gene family analysis

The pan-genome analysis was performed among all 46 assemblies (Supplementary Fig. 12). There were 18,064, 4,859, 19,686, and 4,921 genes identified as core genes, softcore genes, dispensable genes, and private genes, respectively. Functional annotation revealed that 80.20% of the core genes and 56.14% of the softcore genes contained potential functional motif, the percentage of which was higher than the 21.67% of dispensable genes.

Supplementary note 4: Construction of tomato graph pangenome (TGG)

4.1 SNPs and InDels calling from HiFi reads

When mapped the HiFi reads to SL5.0 and called SNPs/InDels with DeepVariant[44], we identified a total of 16,191,489 variants passing all the filters above, consisting of 14,918,816 SNPs and 1,272,673 InDels. We subsequently calculated the genotype heterozygosity rate of each accessions using these SNPs/InDels by plink 2.0 (ref. [45]). The heterozygosity was defined as (N(NM) - O(HOM))/ N(NM), where N(NM) refers to the number of variants observed and where O(HOM) refers to the number of homozygous variants observed. There is a total of 9 accessions with genotype heterozygous rate larger than 2%, and these accessions were retained to represent the diversity of tomato (Table S2).

4.2 SVs

A total of 238,490 SVs called from 100 diverse tomato accessions were downloaded from the official website with the coordinates of SL4.0. These SVs involved 104,791 insertions (INS), 118,863 deletions (DEL), 5,722 duplications (DUP), 2,586 inversions (INV), and 6,528 translocations (TRA). After lifting over to SL5.0 coordinates, a total of 154,725 SVs were retained: 78,155 insertions (INS) and 76,570 deletions (DEL). As SVs were defined as longer than 30-bp in original study, we found that the length of 106,559 SVs (52,626 INSs and 53,933 DELs) was longer than 50 bp. However, we retained all these variants as they represented variations among accessions. For SVs from HiFi reads, as SVs were detected for each accession individually, we firstly normalized SVs for each accession and then merged all SVs, including the SVs from the 100 tomatoes into a dataset with *norm* and *merge* functions implemented in bcftools[46]. This generated a redundant SVs set comprising 364,075 SVs.

4.3 Construction of TGG1.0

To construct TGG1.0, we firstly filtered the problematic near-duplicate variants based on the results of 20 diverse accessions (Table S16) with at 20x short reads. Both deletions with more than 80% reciprocal overlap or insertion distances less than 50 bp from each other and more than 80% reciprocal overlap were considered near-duplicates. Short reads were then mapped to the reference genome, and the support reads for the near-duplicate variants were calculated.

The SVs with the highest count of supported reads were retained in SVs clusters, and finally, we generate a non-redundant SVs dataset. The non-redundant SVs included a total of 217,555 SVs (101,138 INSs and 116,417 DELs). We then normalized and merged the SNPs and InDels called from the HiFi reads with the non-redundant SVs by bcftools[46], resulting a dataset containing 16,387,074 variants (14,927,145 SNPs, 1,263,972 InDels, and 195,957 SVs). These variants were used to construct TGG1.0 with the software vg[47].

4.4 Variant calling with short reads

To call variants for the 706 accessions with short reads sequencing, we indexed TGG1.0 with GBWT based on the greedy path-cover algorithm (n was set 32 for nodes). The minimizer index was set with default minimizer length of 29 and window size of 11. The short reads from each accession were then mapped to TGG1.0 with Giraffe[48] in the output of bam format, and SNPs/InDels were called using DeepVariants[44] with the NGS model. These raw SNPs and InDels were filtered as above. We ultimately called a total of 12,843,097 SNPs and 915,273 InDels with minor allele frequency greater than 0.01.

4.5 Construction of TGG1.1

All the variants used for the construction of TGG1.0 and the SNPs and InDels called from the 706 accessions were integrated and used to construct TGG1.1. As there is no more SVs from 706 accessions, the number of SVs remained the same (195,957) as that in TGG1.0. When merging the SNPs and InDels, we retained the variants in TGG1.0 if the variants from two dataset were not consistence in the same position. A total of 2,971,586 of SNPs and 235,189 InDels were added to the construction of TGG1.1, totaling 17,898,731 SNPs, 1,499,161 InDels, and 195,957 SVs. An example showing the difference of an InDel between TGG1.0 and TGG1.1 is visualised as Supplementary Fig. 13.

Supplementary note 5: Benchmark study of variant calling

5.1 Simulation of short reads

To be as consistent as possible with empirical results, we simulated the variants from the 31 accessions with HiFi reads. Firstly, we simulated the genome with known SNPs using custom scripts and further simulated InDels and SVs using simuG[49] software for each accession. We then simulated short-reads with different coverage (5x, 10x, 15x, 20x, and 25x) from the simulated genome with art_illumina[50] software (-ss HS20 -m 500 -s 10 -l 100).

5.2 Evaluation

Like we did for the real dataset, we evaluated the performance of linear reference genome and graph pangenome on variant calling for all categories (SNPs, InDels, and SVs). We constructed the graph pangenome using vg by taking all known variants from the 31 accessions used for simulation. The SNPs and InDels were called using vg and the SVs were genotyped using paragraph of different coverage levels of short reads. For linear genome, we called the SNPs and InDels using the bwa and DeepVariant pipeline and SVs were called as described above. The performance of variant calling one SNPs and InDels were evaluated using the software hap.py (https://github.com/Illumina/hap.py) and performance of SVs calling were evaluated using the software truvari[51]. The results showed that the graph pangenome was better than the linear reference genome for all the three categories of variants (Table S6).

5.3 Comparison of SNPs and InDels from SL5.0 and TGG1.1

To evaluate the correctness of variant calling of TGG1.1 and SL5.0, we compared the SNPs and InDels called by the two methods. In total, 759,820 InDels and 12,117,887 SNPs were detected by both methods; 1,294,175 SNPs and 139,207 InDels uniquely detected by SL5.0, and 725,210 SNPs and 155,453 InDels uniquely detected by TGG1.1. We evaluated the depth distribution of these uniquely detected variants and found that the depth of SL5.0 uniqely detected variants was the same as that of these overlapped variants, while the depth of TGG1.1 uniquely detected variants was slightly lower than that of the overlapped variants. Furthermore, we noticed that we could retrive approximately 74% of SL5.0 uniquely detected variants, which were filtered due to poor quality or low depth, indicating that the quality filter might be too

strict to filter some true positive for TGG1.1. However, it is difficult to choose a perfect parameter set to balance the false positives and false negatives, as we do not have a true dataset for benchmarking.

We further compared the two callsets with a validated SNPs chip, which contains validated 7720 SNPs[52]. We notice that there exits 394 SNPs uniquely deteced by graph pangenome, and there are more SVs nearyby these SNPs (Supplementary Fig. 14), indicating that the better mapping quality in these regions using graph pangenome.

Supplementary note 6: Heritability analysis

6.1 Phenotype simulation

To choose a suitable parameter for our study and evaluate the impact of incomplete LD on the estimation of heritability, we simulated multiple scenarios with different numbers of quantitative trait nucleotides (QTNs; 10, 100, 500, and 1000) and heritability levels (0.15, 0.30, 0.45, 0.60, 0.75, and 0.90). QTNs were simulated from SNPs and InDels or from SVs. Overall, a total of 48 combinations were simulated using LDAK[53] software with other parameters were set as default. Each combination was repeatedly simulated 100 times.

6.2 Effect of the parameters

To select a proper value for the parameter of power, we estimated the heritability under different values of power ranging from -1 to 0, with an increase of 0.1. Heritability was estimated using the dataset used for simulation. We found that the power of -0.5 gives the best estimations under most scenarios (Supplementary Fig. 15). In addition, we compared the alternative likelihood of heritability generated by LDAK, and we found that the likelihood under power of -1 was not significantly higher than that under the power of -0.5 (Supplementary Fig. 16), therefore, the power of -0.5 was used in our study.

6.3 Effect of incomplete LD

To evaluate the effect of incomplete LD on heritability estimation, we compared the heritability estimation with or without simulated QTNs. That is, we simulated the phenotype with QTNs from SNPs and InDels and estimated the heritability using all SNPs and InDels (including the QTNs) or estimated the hereditability using all SVs (excluding the QTNs). Alternatively, we simulated the phenotype with QTNs from SVs and estimated the heritability using all SVs (including the QTNs) or estimated the hereditability using all SNPs and InDels (excluding the QTNs). The results showed when QTNs were included in the dataset, the estimated heritability is approximately the same as the simulated heritability; however, when QTNs were not included in the dataset, the heritability was underestimated (Supplementary Fig. 2).

6.4 The effect of power

To select a proper value for the parameter of power, we estimated the heritability under different values of power ranging from -1 to 0, with an increase of 0.01. Heritability was estimated using the dataset used for simulation. We found that the power of -0.5 gives the best estimations under almost all scenarios (Supplementary Fig. 15). In addition, we compared the alternative likelihood of heritability generated by LDAK, and we found that the likelihood under power of -1 was not significantly higher than that under the power of -0.5 (Supplementary Fig. 16), therefore the power of -0.5 was used in our study.

6.5 Heritability of *cis* regions

To estimate the heritability of cis regions, the variants were divided into six categories, namely, the *cis* SNPs, *cis* InDels, *cis* SVs, *trans* SNPs, *trans* InDels, and *trans* SVs. We derived the kinship matrix for each category and the heritability was estimated with a total of six random effects in a composite model using LDAK-thin model. The *cis* variants were defined as those variants located within 50 kb either up- or down-stream of a gene. All the remaining variants were taken as *trans* variants.

6.5 Heritability of leading variants

To estimate the heritability of significant variants in GWAS, we picked the leading significant variant within a QTL and derived a kinship matrix with this leading variant. The heritability was estimated for each leading variants using LDAK software.

6.5 Heritability of local variants

To estimate heritability of local variants, we picked up those variants within 50 kb on each side of the leading significant variants, and derived kinship matrix with SNPs, InDels, and SVs, respectively, and the heritability was estimated with a total of three random effects in a composite model using LDAK-thin model.

Supplementary note 7: Co-expression network analysis

Following the above methods, the 19,353 expressed genes (17,785 SL5.0 genes and 1,568 non-reference genes) were assigned to 99 modules (Supplementary Figs. 17 and S18). When only the 17,785 SL5.0 genes were analysed, there were only 86 modules assigned, indicating the importance of non-reference genes in detecting modules. In addition, for the 99 modules identified, we found that non-reference genes were involved in 81 modules, of which 11 were served as hub genes in the corresponding modules.

To visualize the overall co-expression network, Gephi (v0.9.2)[54] software was used. In the visualizations, the nodes represented genes and edges represented the correlation coefficients of two connected genes.

This module was identified through the identification of *SlMYB12*, as this gene has been studied and is known to affect the content of flavonoids. A total of 81 genes were identified in this module. Furthermore, we annotated these genes based on Kyoto Encyclopedia of Genes and Genomes (KEGG) pathways and found 7 genes related to flavonoid biosynthesis. Two genes encode CHI or F3H enzymes whose catalytic activity yields the naringenin or that uses naringenin as a substrate. As these two genes are directly involved in the biosynthesis of naringenin, we considered these two genes as core genes based on the definition of omnigenic model.

Supplementary note 8: a web-based tomato database

Solomics tomato database is a comprehensive multi-omics database for better tomato breeding. The web-based database contains an integrated collection of genomic data from 838 tomato genomes and a phenotypic collection of 19,353 expression traits and 970 metabolite traits. Notably, a high-quality new built tomato graph pangenome (TGG1.1) is available in the database, it contains millions of identified genetic variants including SNPs, InDels and SVs that could be interesting to both molecular biologists and breeders for research and application in practice.

The website provides several tools to interact with the database, popular tools such as BLAST, JBrowser, and Primer 3 are embedded on the website. The website has following features. There are some key features of our database:

CRISPR database. For users' convenience, we have pre-calculated the sgRNA targets for each annotated gene using CRISPR_local pipeline for all assemblies.

KASP marker design. The database provides a comprehensive collection of SNPs, InDels and SVs identified from the tomato graph pangenome that could be used as candidates for MAS, the identified variants in the database can serve as rich recourses for primer design studies. For molecular breeders, the website provides tools to design KASP markers.

GWAS results. A fully detailed summary statistics of GWAS results of all the identified genetic variants are available in the database, allowing users to search directly for future research.

Supplementary note 9: DNA capture array of SVs

9.1 Marker selection for SVs array panel

To select SVs for DNA capture array, we selected SVs as following: Firstly, SVs located in the proximal region of annotated genes regions were selected. Then, the selected SVs should not in complete LD (SVs with weighted value large than zero from LDAK-thin procedure). Next, we excluded these SVs that were missing in at least 60% individuals among the 332 accession and these variants with MAF less than 0.05. Finally, we kept all SVs that were significant associated with any molecular traits, regardless of whether they passed the filters above or not. The distribution of final SVs sets is represented in Supplementary Fig. 19 and the data is available at website http://solomics.agis.org.cn/tomato/ftp/.

9.2 Conceptual design of probes for tomato SV gene panel

In the process of probe design, to prevent mutations at breakpoints of SVs from affecting the capture efficiency of probes, we can use the "flank compensation" approach. Flank compensation refers to a situation where areas of the genome is too complex, we do not design the probe for the target area directly, but for the flank area of the target area. As described in Supplementary Fig. 20a, in capture sequencing results, a normal distribution of sequencing depth is expected surrounding the area where probe is designed. The sequencing area is obtained not only for the probe design area, but also for the flank area of the target area.

For the three different SVs (insertion, deletion, and inversion) and different variant lengths, we use different probe design strategies. For insertions, we first “fill” the insertion into the reference genome to form a larger pseudo-genome (Supplementary Fig. 20b). The probe design is then carried out depending on the length of insertions (Supplementary Fig. 20c). When the insertion length is shorter than 120-bp, only one probe covering the variation area flanking both sides will be picked. For insertions with lengths from 120-bp to 300-bp, two probes on both flanks of variant area and two probes on the flanks and one in the middle of the variant will be picked if longer than 300-bp. For deletions, probes can be designed directly on reference genome (Supplementary Fig. 20d). One probe covering the area or two probes on both flanks of the variants will be picked based on inversion length (Supplementary Fig. 20e). The genotyping of variants will be inferred from the read depth (Supplementary Fig. 20f).

Supplementary figures

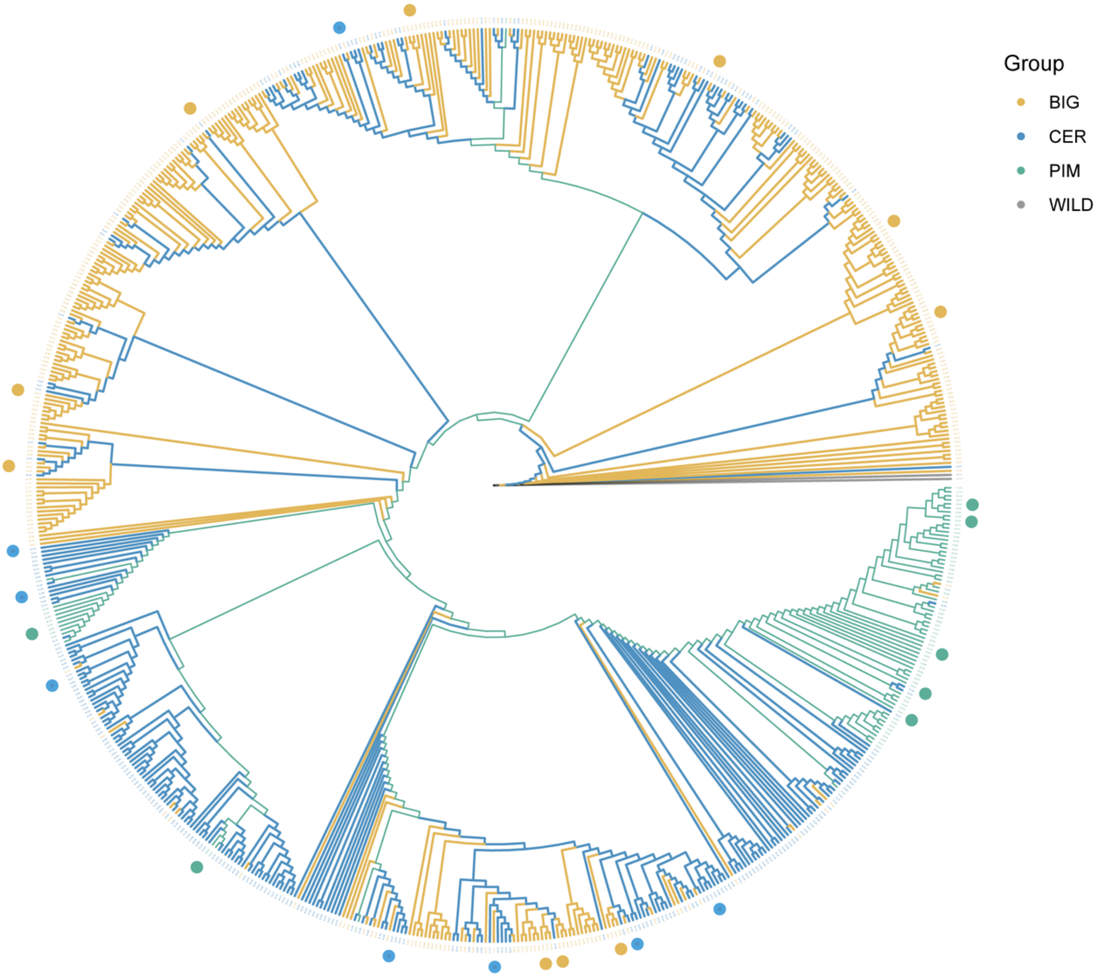

Supplementary Fig. 1 | Phylogenic tree of 706 accessions. The phylogenetic tree is constructed with randomly chosen 100K SNPs. Two wild tomato accessions are used as the outgroup. Colored dots indicate a subset of the 24 selected for HiFi sequencing. RAxML[55] software is applied with 100-times bootstrap and GTRGAMMA model parameters.

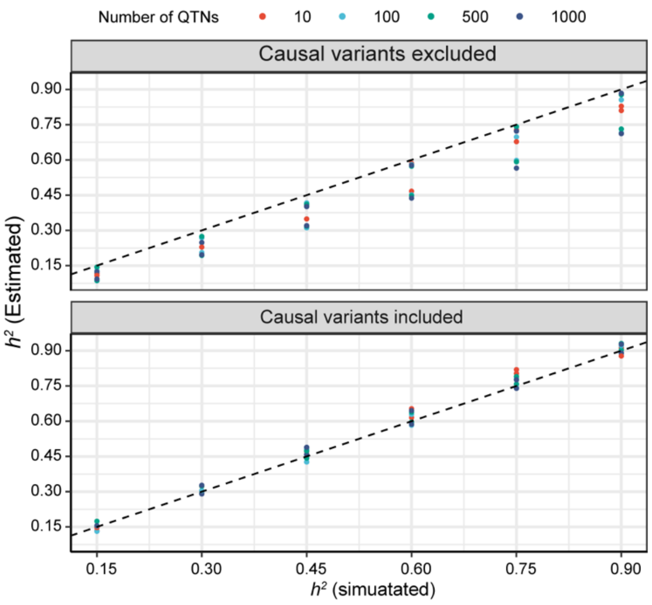

Supplementary Fig. 2 | Simulation study to evaluate the impact of incomplete LD for heritability. The scenarios simulate 10, 100, 500 and 1000 causal QTNs with five heritability levels (0.15, 0.30, 0.45, 0.60, 0.75, and 0.90). Each combination is repeatedly simulated 100 times.

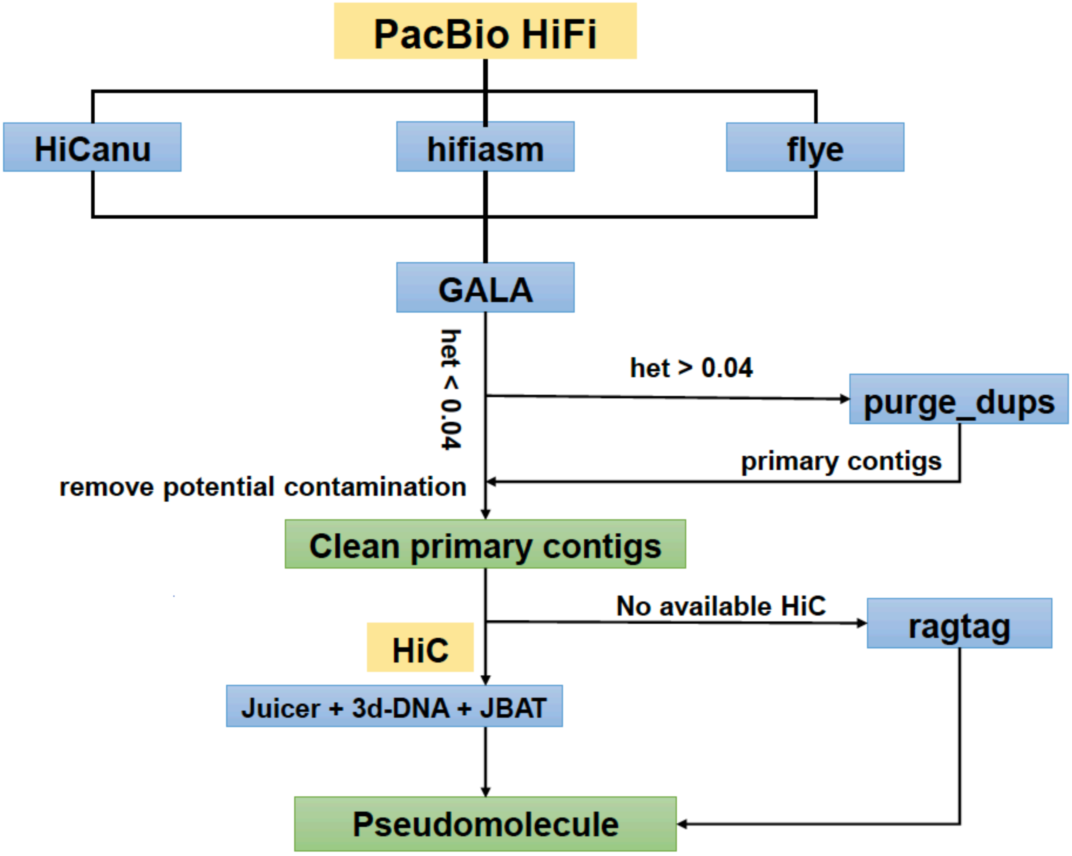

Supplementary Fig. 3 | Pipeline of genome assembly and scaffolding. The results of three assembly software are integrated to one clean primary contigs which subsequently scaffold by HiC data or according to reference-guided methods. Blue box: the software used in the process; yellow box: input datasests; green box: results from previous step.

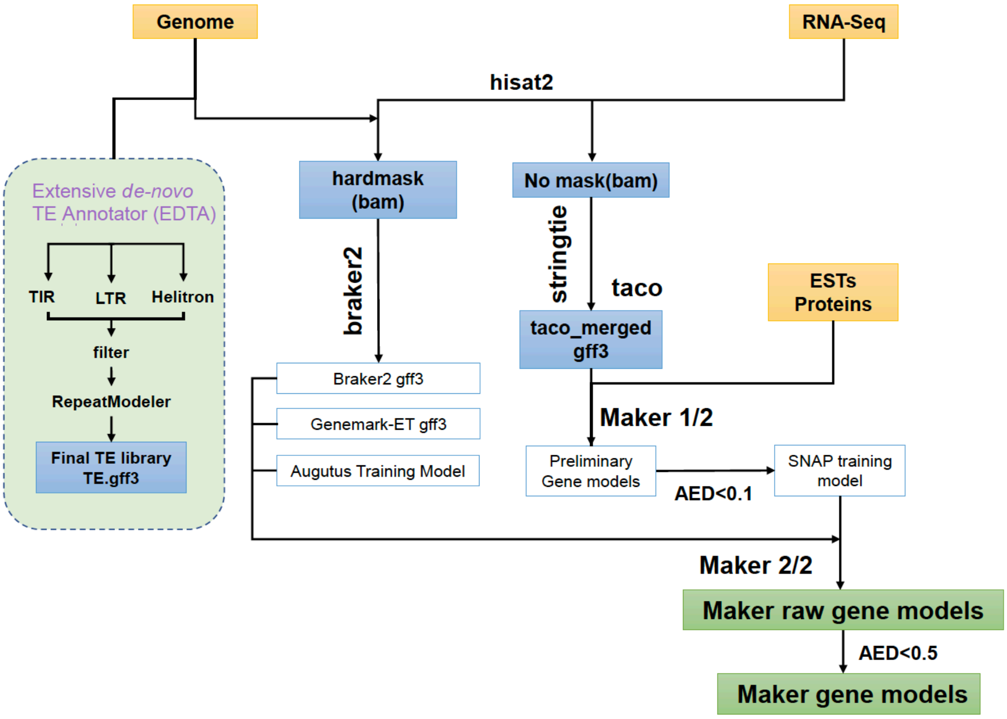

Supplementary Fig. 4 | Overview of the genome annotation pipeline. The EDTA pipeline is used for the annotation of TEs. The masked genome is as input data for ab initio prediction. The public ESTs are as evidence for homology-based prediction. Finally, MAKER is applied to integrate above prediction results.

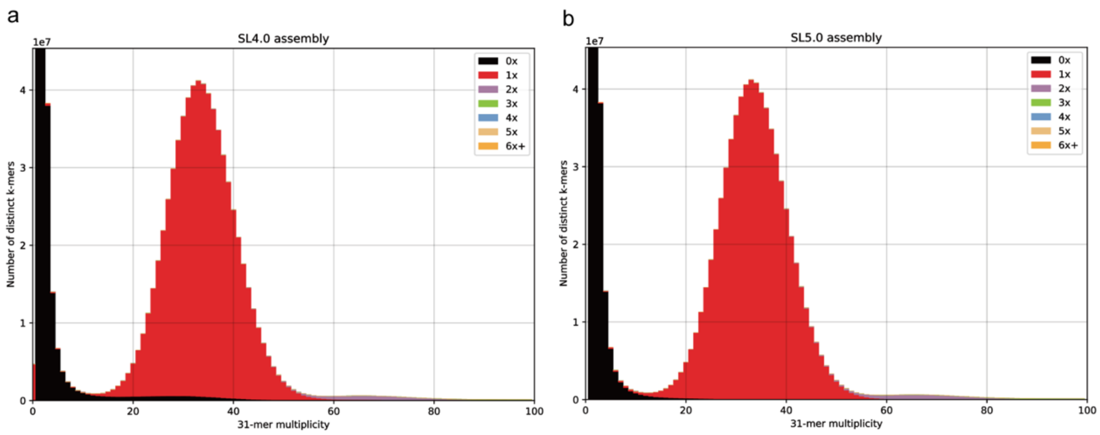

Supplementary Fig. 5 | K-mer spectrums of SL4.0 and SL5.0. Comparison of 31-mer spectra between frequency of occurrence (x-axis) and number of distinct k-mers (y-axis). The missing k-mers depict black; k-mers appear once depicted red; purple represents twice and etc. The k-mer is set to 31. The missing k-mer distribution of SL5.0 between k-mer multiplicity from 15 to 50 is lower than SL4.0.

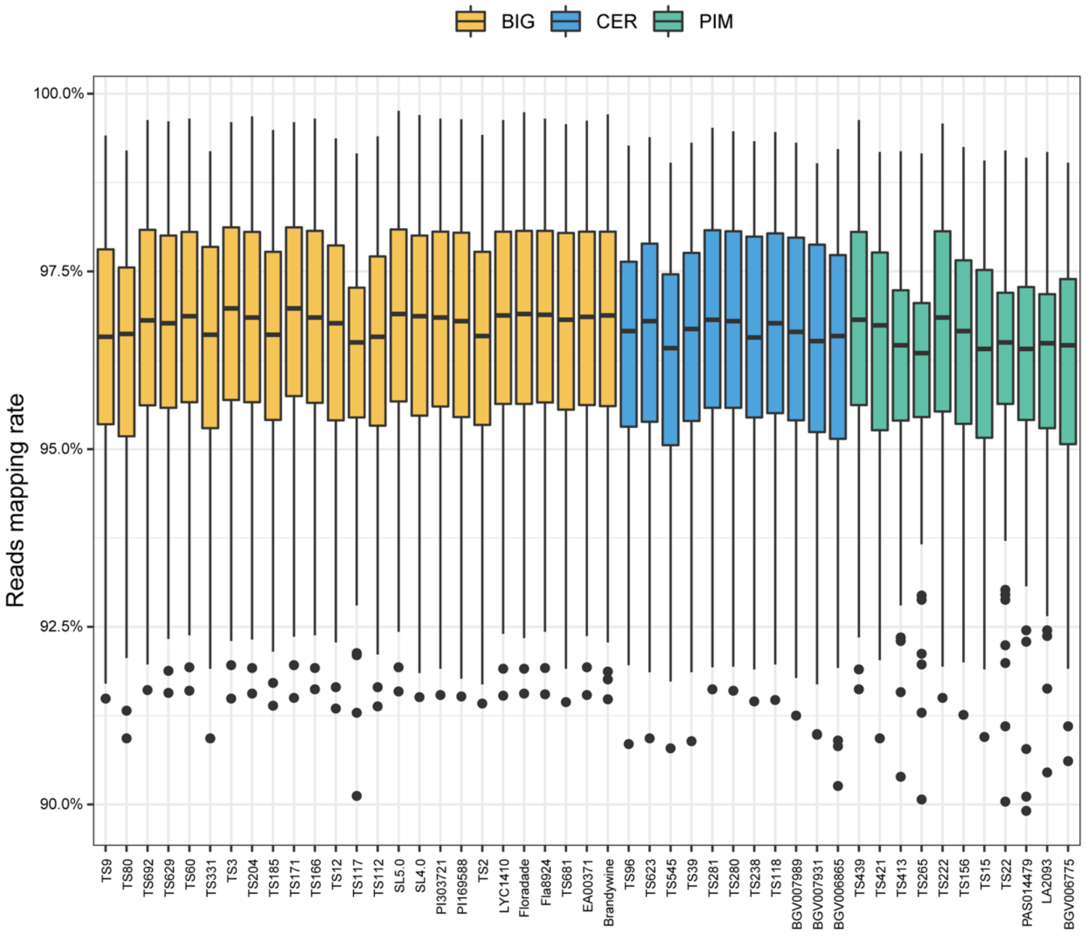

Supplementary Fig. 6 | Mapping percentages of RNA-seq reads across 46 assemblies. The x-aixs represents corresponding accessions and the y axis represents 83 public SRA reads genome mapping rate. Box and whisker plots with centre line = median, cross = mean, box limits = upper and lower quartiles, whiskers = 1.5 × interquartile range and solid points = outliers. n = 83 representative public RNA-seq data were analysed.

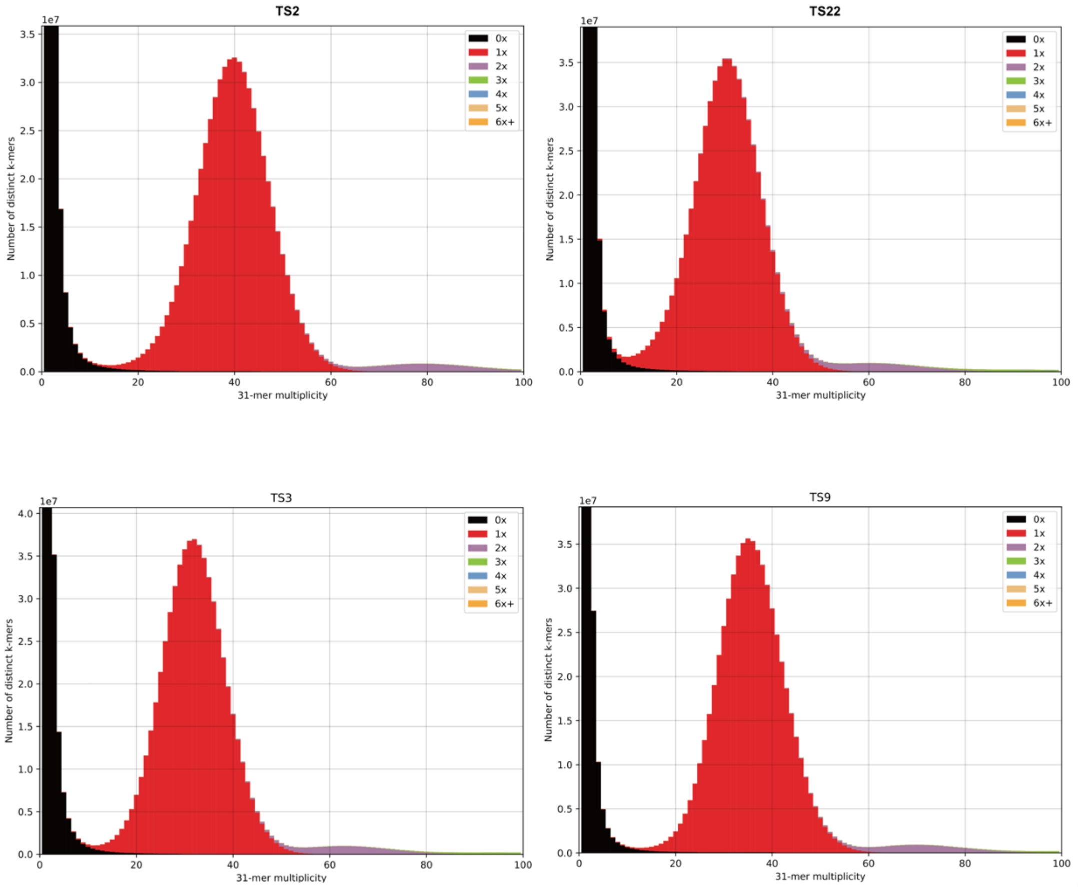
TS2
TS22
TS3
TS9
Number of distinct k-mers
31-mer multiplicity
1e7
0x
1x
2x
3x
4x
5x
6x+

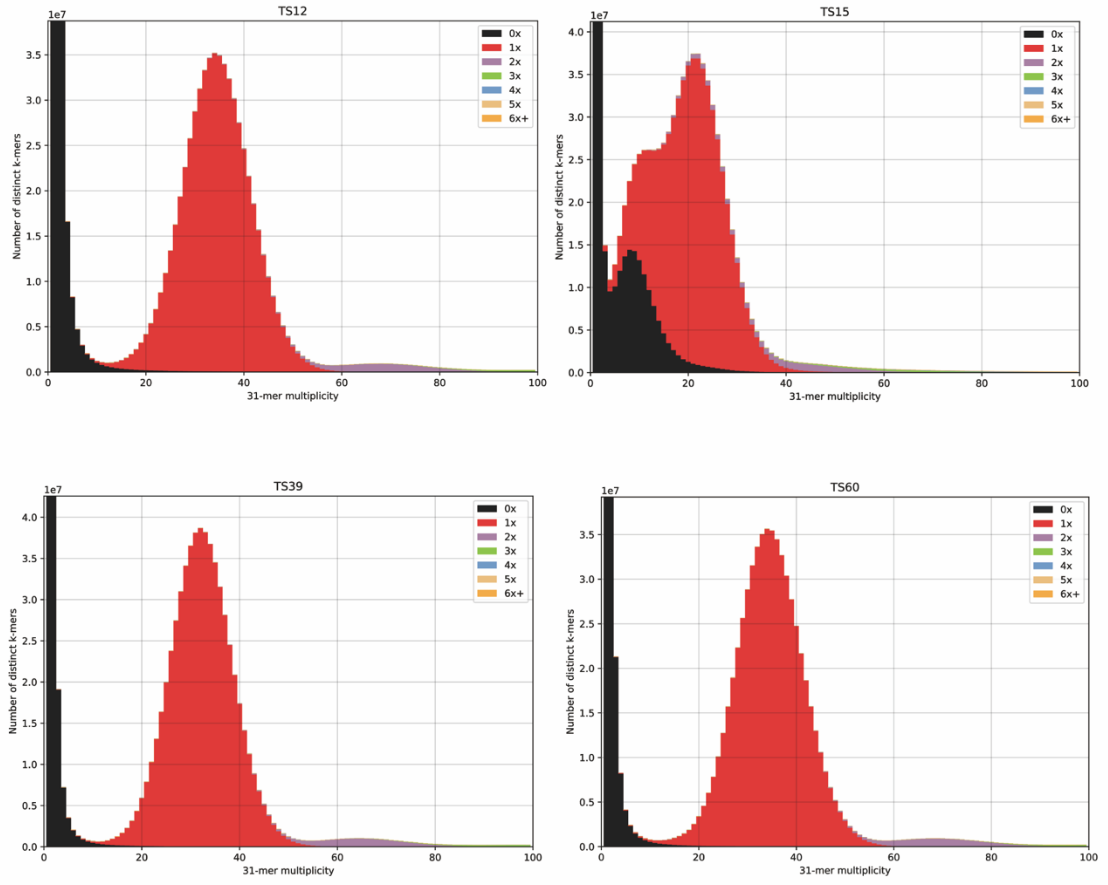
TS12
TS15
TS39
TS60
1e7
Number of distinct k-mers
31-mer multiplicity
0x
1x
2x
3x
4x
5x
6x+

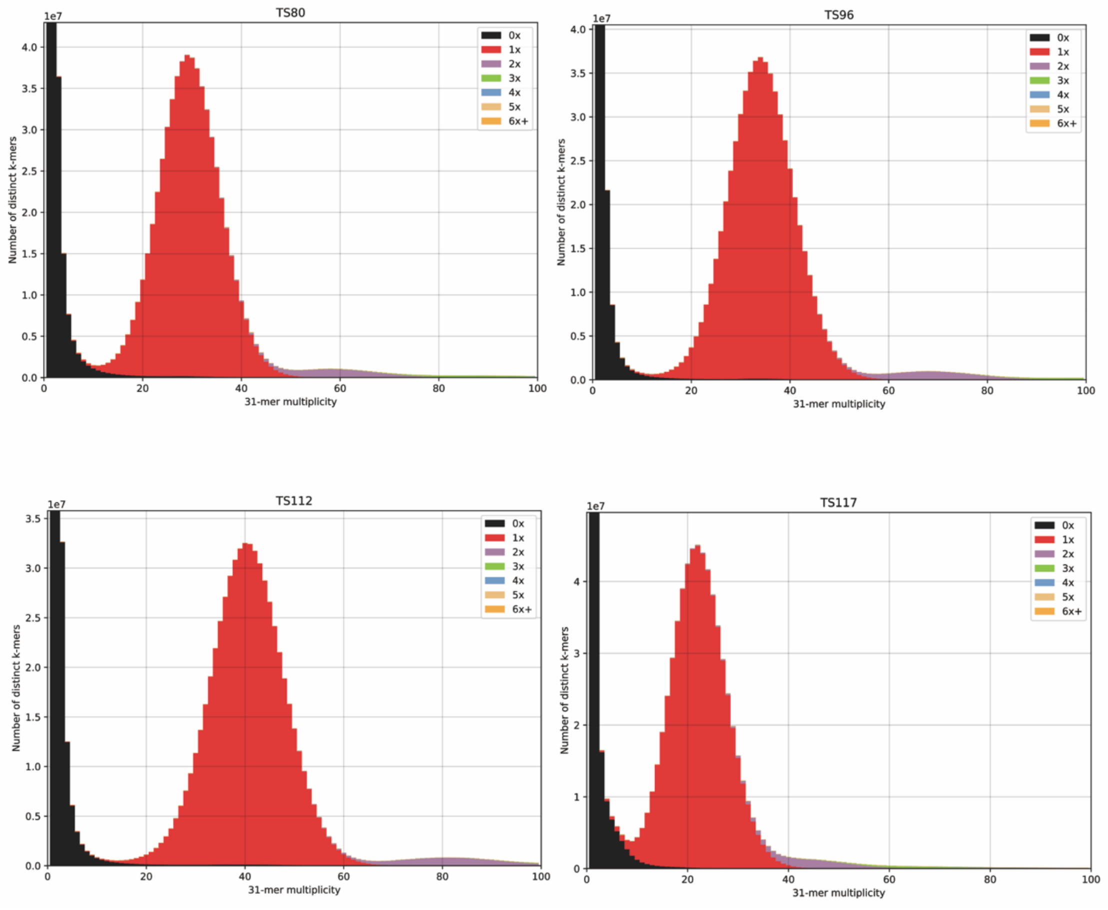
TS80
TS96
TS112
TS117
1e7
Number of distinct k-mers
31-mer multiplicity
0x
1x
2x
3x
4x
5x
6x+

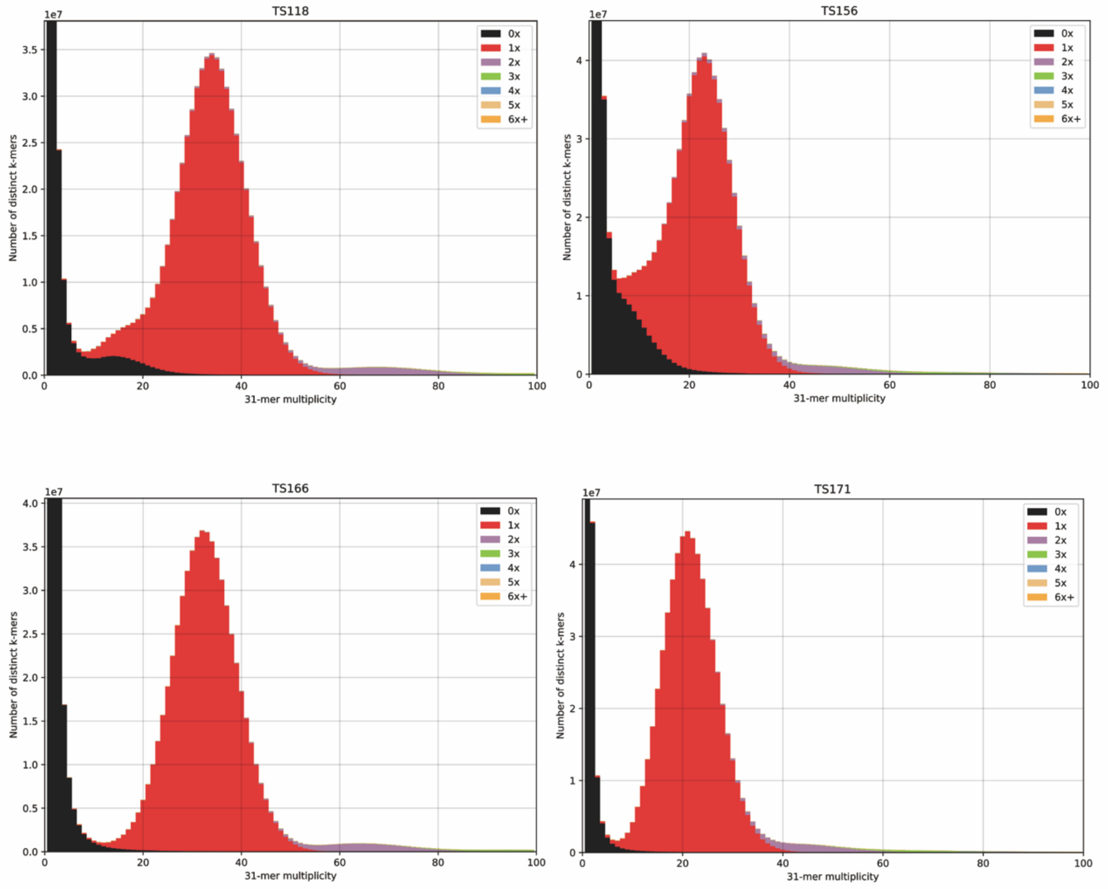
TS118
TS156
TS166
TS171
1e7
Number of distinct k-mers
31-mer multiplicity
0x
1x
2x
3x
4x
5x
6x+

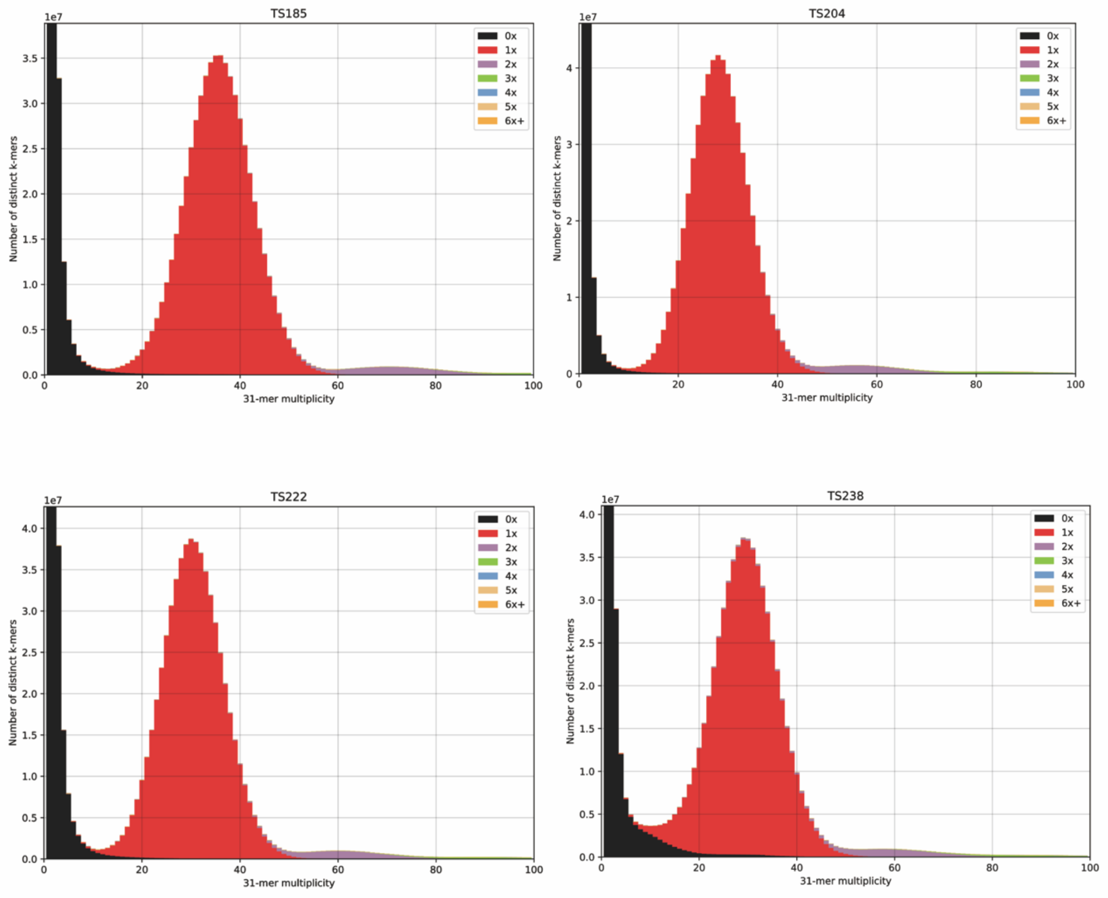
TS185
TS204
TS222
TS238
1e7
Number of distinct k-mers
31-mer multiplicity
0x
1x
2x
3x
4x
5x
6x+

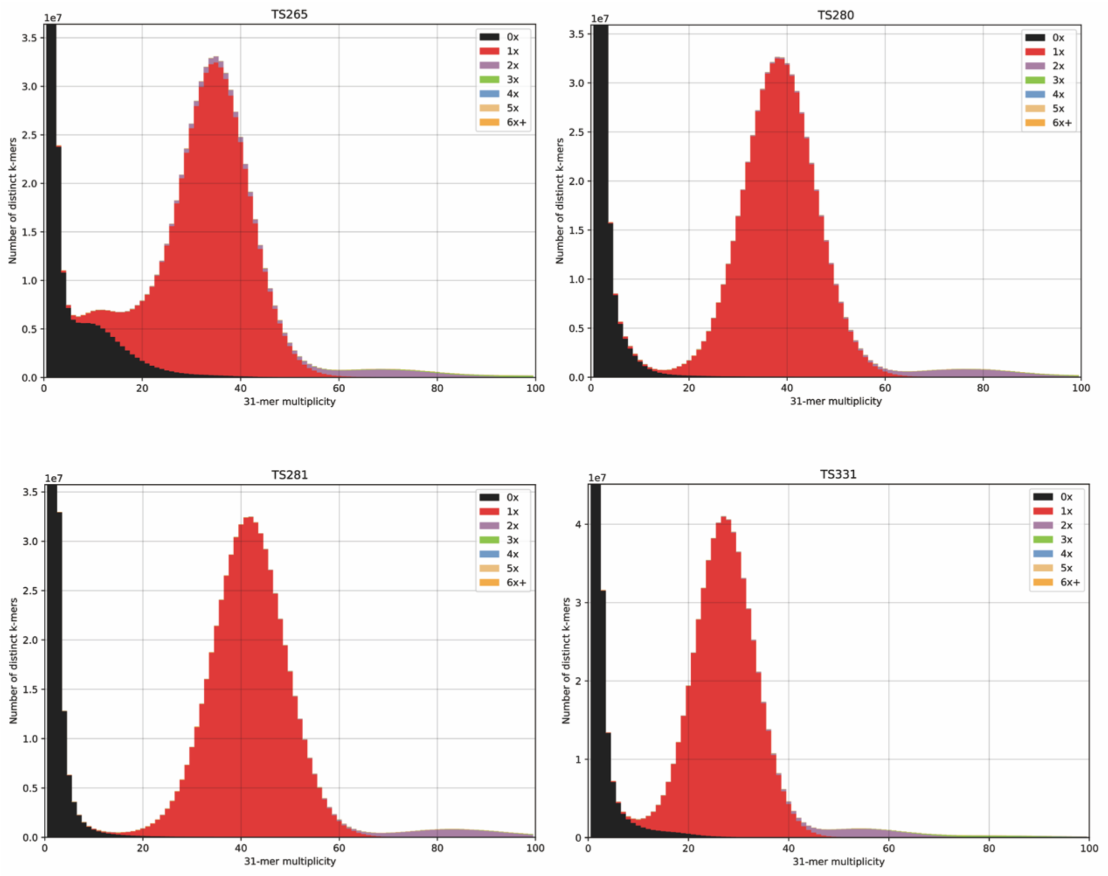
TS265
TS280
TS281
TS331
1e7
Number of distinct k-mers
31-mer multiplicity
0x
1x
2x
3x
4x
5x
6x+

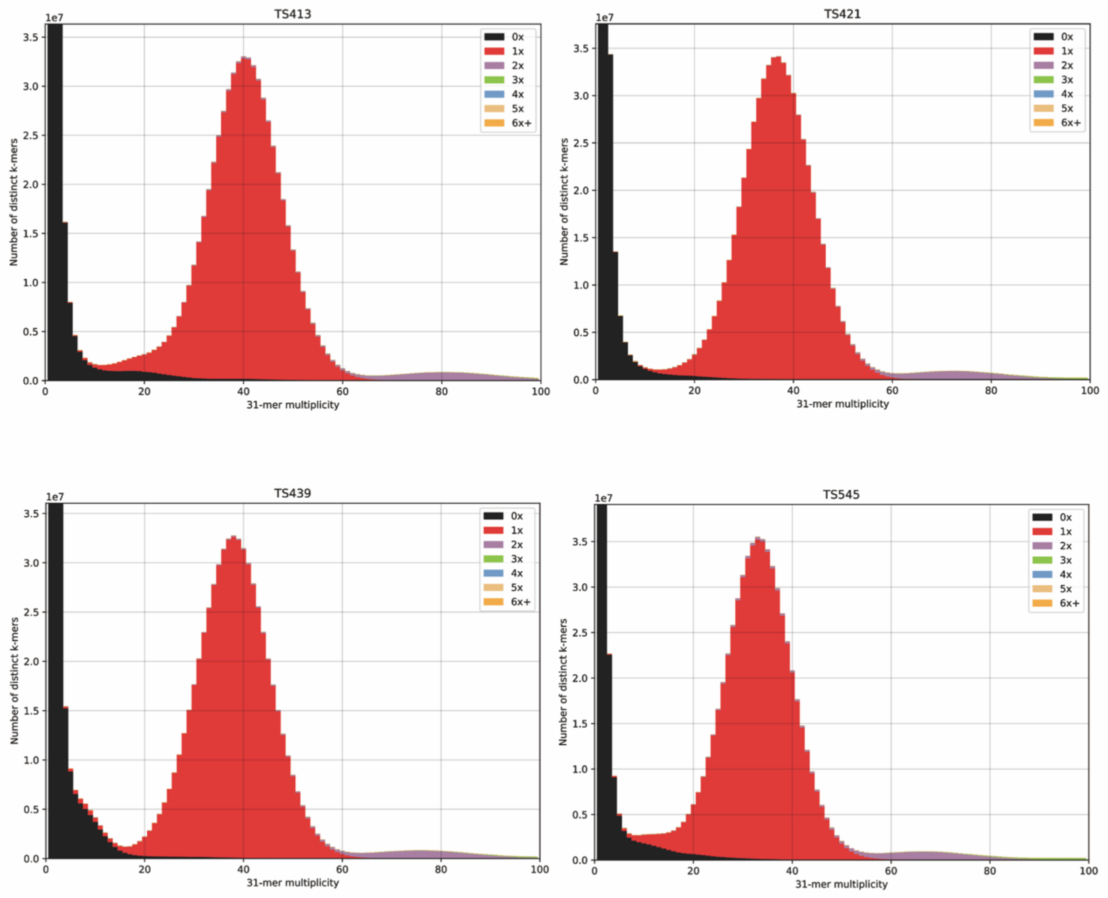
TS413
TS421
TS439
TS545
Number of distinct k-mers
31-mer multiplicity
1e7
0x
1x
2x
3x
4x
5x
6x+

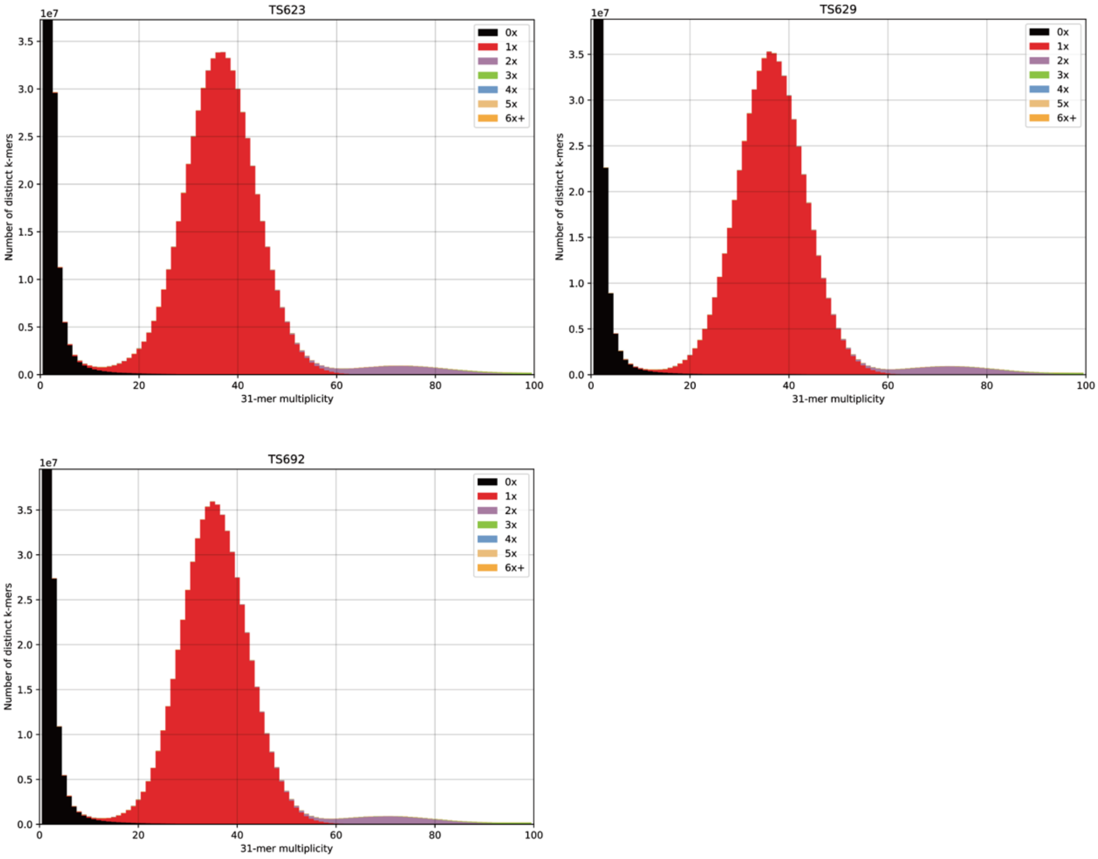

Supplementary Fig. 7 | The KAT spectrums for each assembly from HiFi reads. Comparison of 31-mer spectra between frequency of occurrence (x-axis) and number of distinct k-mers (y-axis) in 31 assemblies. The missing k-mers depict black; k-mers appear once depicted red; purple represents twice and etc. The k-mer is set to 31.

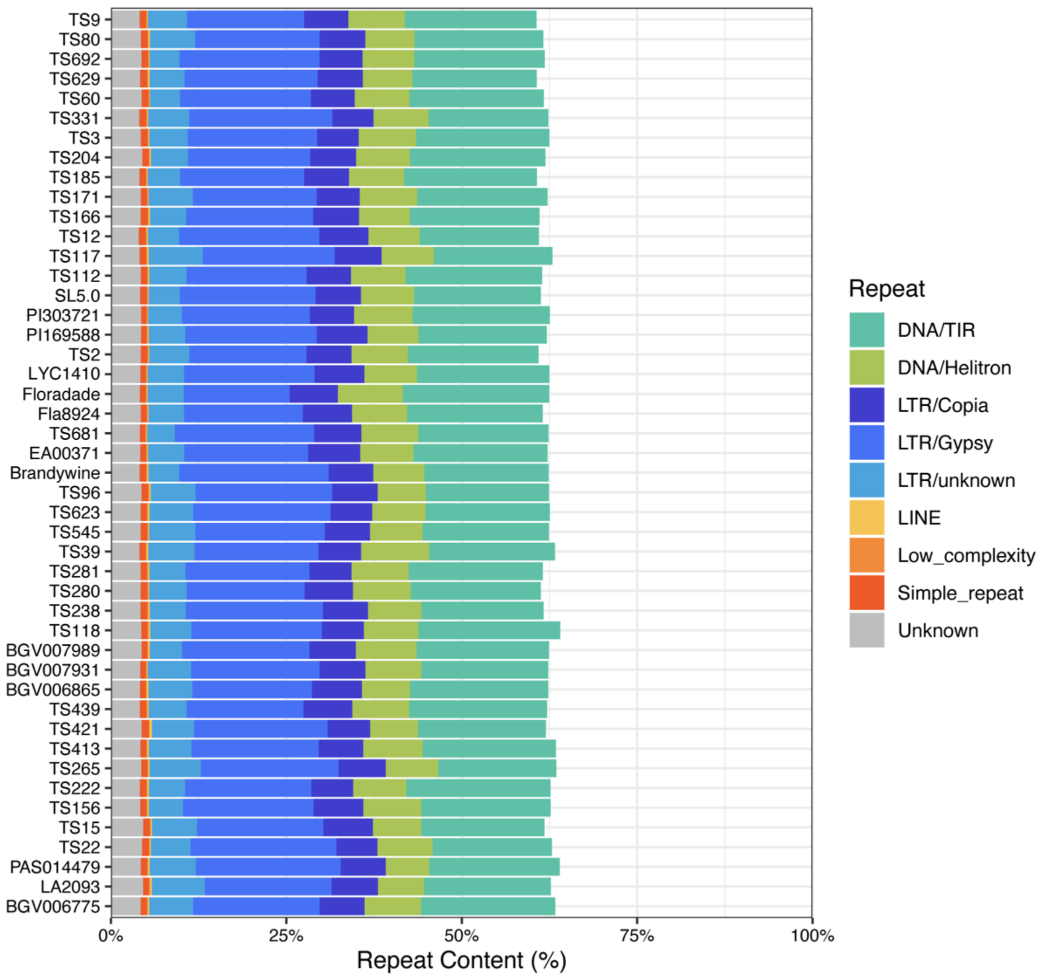

Supplementary Fig. 8 | The cumulative length of repetitive elements for all assemblies. The x-axis represents corresponding repetitive sequence percentage of genome. Each type of repeats is represented by its corresponding colour. Genes and low-copy intergenic regions make up the rest of the assembly.

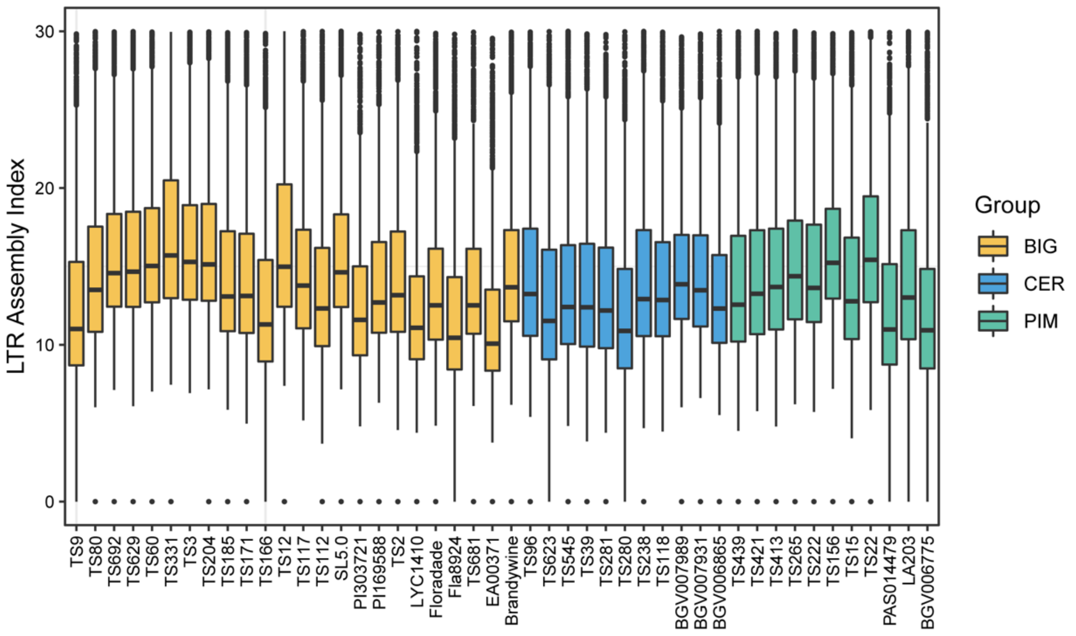

Supplementary Fig. 9 | Assessing of assembly quality using LTR assembly index. The x-axis represents corresponding accessions and y-axis represents LTR Assembly Index (LAI) value. Box and whisker plots with centre line = median, cross = mean, box limits = upper and lower quartiles, whiskers = 1.5 × interquartile range and solid points = outliers. The LAI was calculated according to 3 Mb window and 300 kb step across whole genome. The sample sizes are listed as following: SL5.0 (n = 2,567), TS2 (n = 2,601), TS3 (n = 2,590), TS331 (n = 2,682), TS9 (n = 2,592), TS204 (n = 2,583), TS117 (n = 2,646), TS166 (n = 2,595), TS629 (n = 2,607), TS171 (n = 2,565), TS12 (n = 2,646), TS80 (n = 2,620), TS60 (n = 2,560), TS112 (n = 2,591), TS185 (n = 2,599), TS692 (n = 2,581), TS280 (n = 2,610), TS545 (n = 2,599), TS39 (n = 2,664), TS96 (n = 2,601), TS118 (n = 2,603), TS281 (n = 2,603), TS238 (n = 2,586), TS623 (n = 2,631), TS222 (n = 2,595), TS15 (n = 2,577), TS439 (n = 2,582), TS156 (n = 2,614), TS265 (n = 2,689), TS421 (n = 2,633), TS413 (n = 2,686), TS22 (n = 2,654), LA2093 (n = 2,593), PAS014479 (n = 2,527), BGV006775 (n = 2,529), BGV006865 (n = 2,513), BGV007989 (n = 2,530), BGV007931 (n = 2,495), Brandywine (n = 2,503), TS212 (n = 2,511), TS667 (n = 2,493), TS697 (n = 2,488), TS681 (n = 2,472), TS685 (n = 2,482), TS698 (n = 2,492), Fla8924 (n = 2,519).

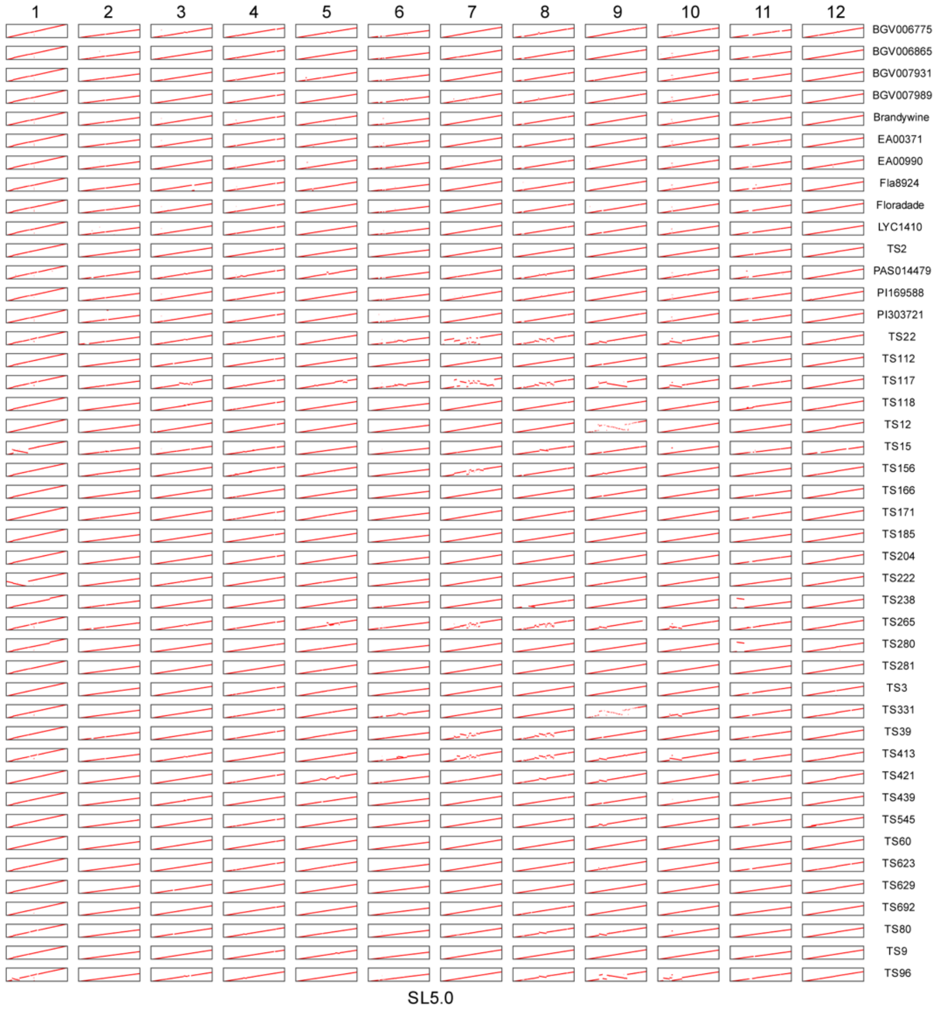

Supplementary Fig. 10 | Collinearity of other assemblies with SL5.0. The x-axis represents the separate 12 chromosome against with corresponding accessions.

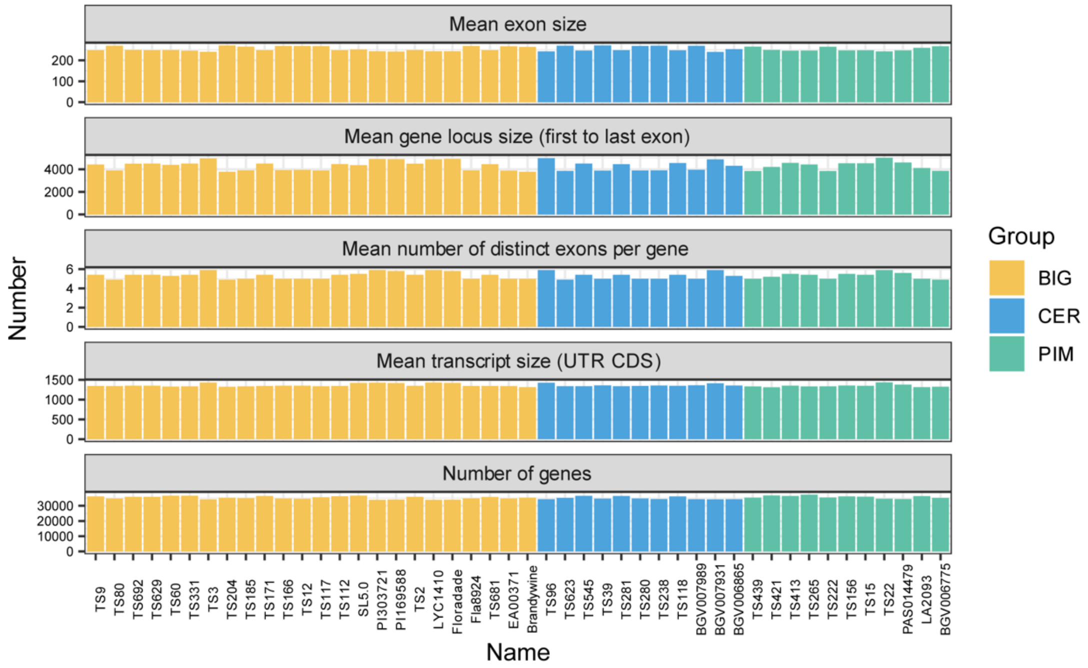

Supplementary Fig. 11 | Characteristic of annotated protein-coding genes among 46 tomato assemblies. The panels represent annotation gene information statistics of the mean of exon size, gene locus size, number of distinct exons per gene, transcript size and the counts of gene from top to bottom in 46 assemblies.

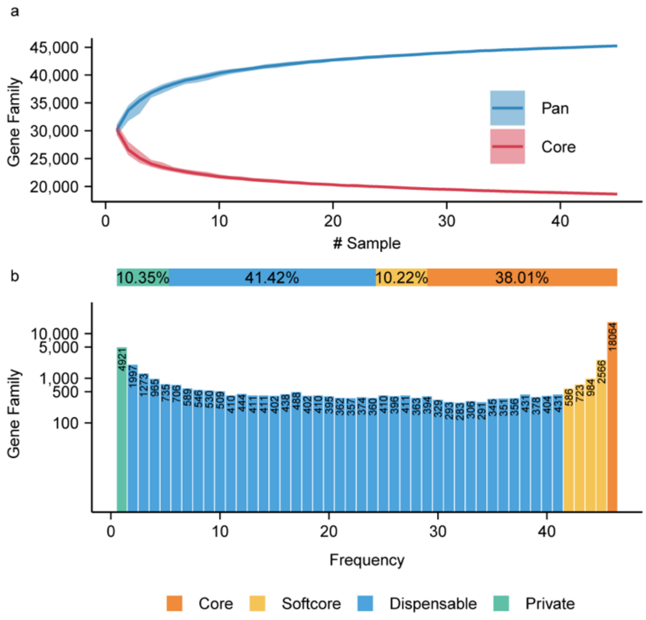

Supplementary Fig. 12 | Pan-genome of tomato. a) Variation of gene families in the pan-genome and core genome along with additional tomato assemblies. Upper and lower edges of the blue and red areas indicate the 5% and 95% intervals of number of gene families. **b)** Compositions of the pan-genome and individual genomes. The "core" genes are presented in all accessions; the "soft core" genes are presented in more than 90% accessions (42 to 45); the "dispensable" genes are presented in at least 2 accessions (2 to 41); the "private" genes are only presented in one accession containing unassigned genes.

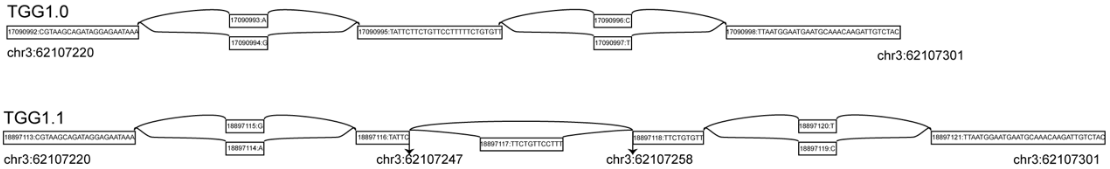

Supplementary Fig. 13 | The visualisation of TGG1.0 and TGG1.1. The 12-bp deletion at Chr3:62107247 is specifically identified in TGG1.1.

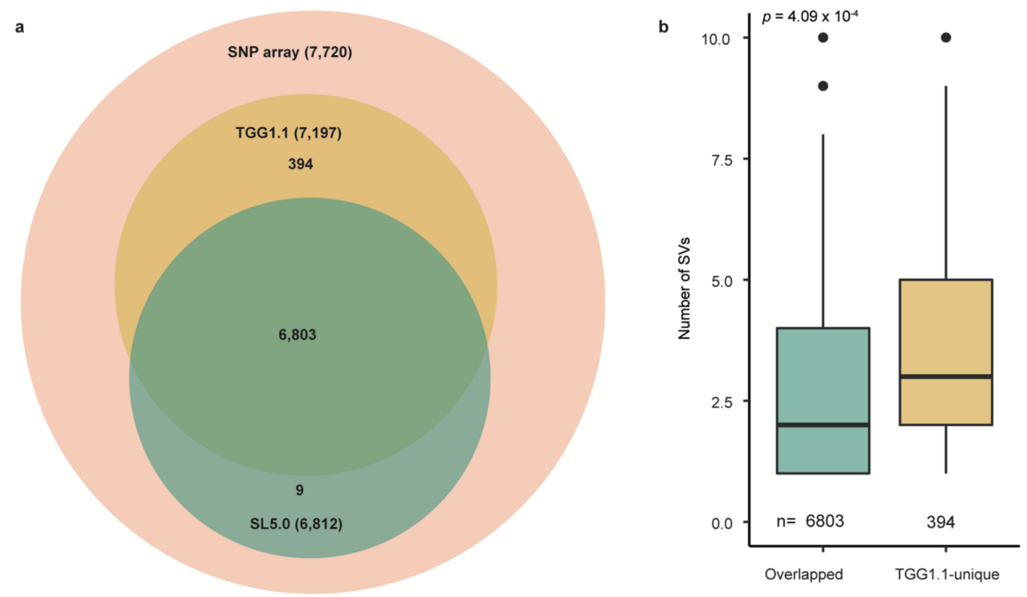

Supplementary Fig. 14 | Compare the validation of SNPs chip between TGG1.1 and linear SL5.0. a) SNPs chip identified overlap or unique in two datasets. **b**) Comparation of SVs counts around 5-kb SNPs chip found in TGG1.1 overlap or unique. Box and whisker plots with centre line = median, cross = mean, box limits = upper and lower quartiles, whiskers = 1.5 × interquartile range and solid points = outliers. *P* values were derived from Wilcoxon tests (two-sided). n represents the sample size.

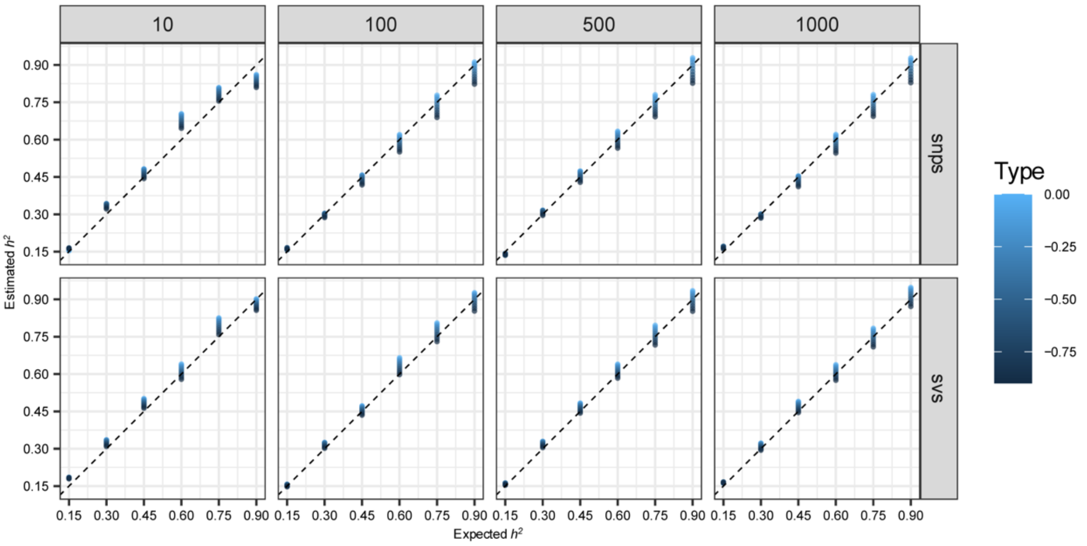

Supplementary Fig. 15 | Simulation studies on evaluation of the effects of different parameters. The number in top of panel refers to the number of QTNs simulated. The top panel represents the results estimated using SNPs and the bottom panel represents the results estimated using SVs. Type refers to different power values used for estimation in LDAK-thin model.

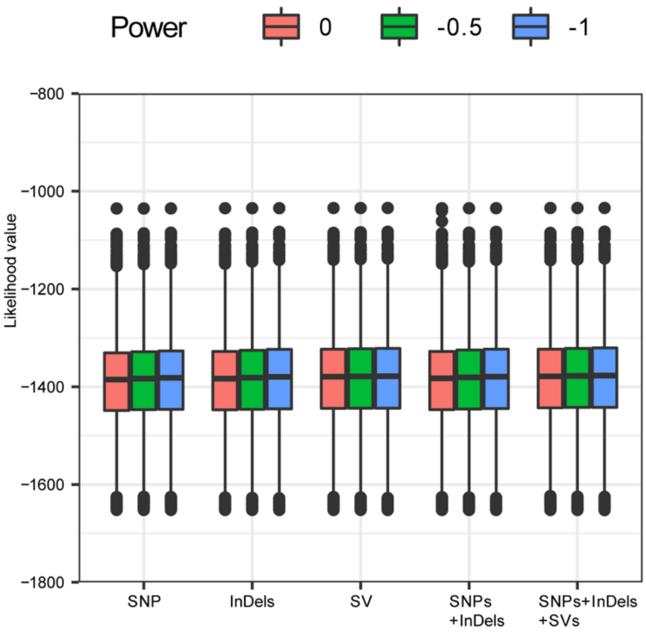

Supplementary Fig. 16 | Likelihood distribution with different values of power. The x-axis represents various genetic markers combinations against with the y-axis which represents likelihood of heritability generated by LDAK. Box and whisker plots with centre line = median, cross = mean, box limits = upper and lower quartiles, whiskers = 1.5 × interquartile range and solid points = outliers. n = 20,353 traits were evaluated.

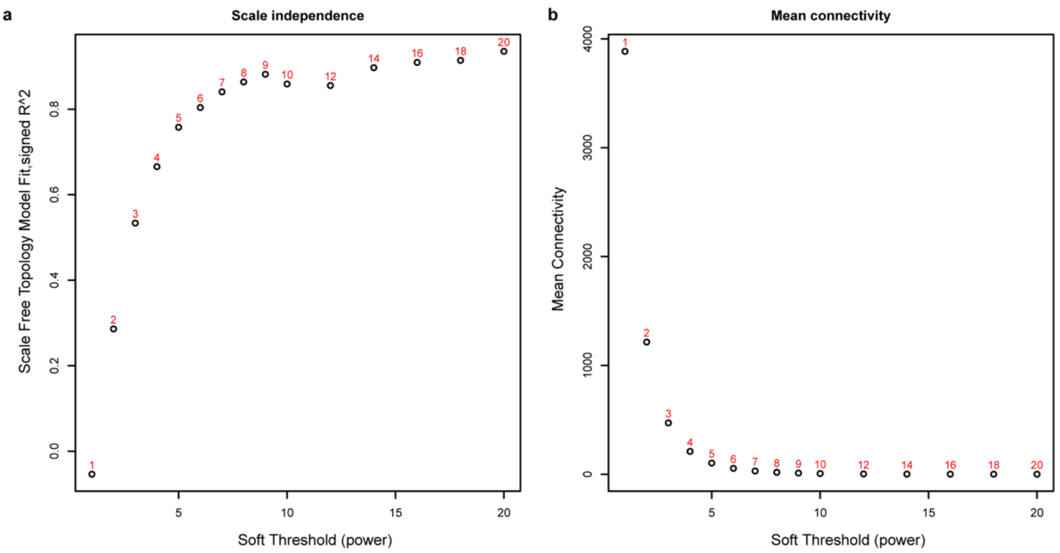

Supplementary Fig. 17 | The parameter, soft threshold, determination for module construction. a) The scale-free fit index versus soft-threshold. **b)** The mean connectivity versus soft-threshold. The fit index curve flattens out upon reaching a high value (>0.9) at the power of 9. This value is selected in the construction of module.

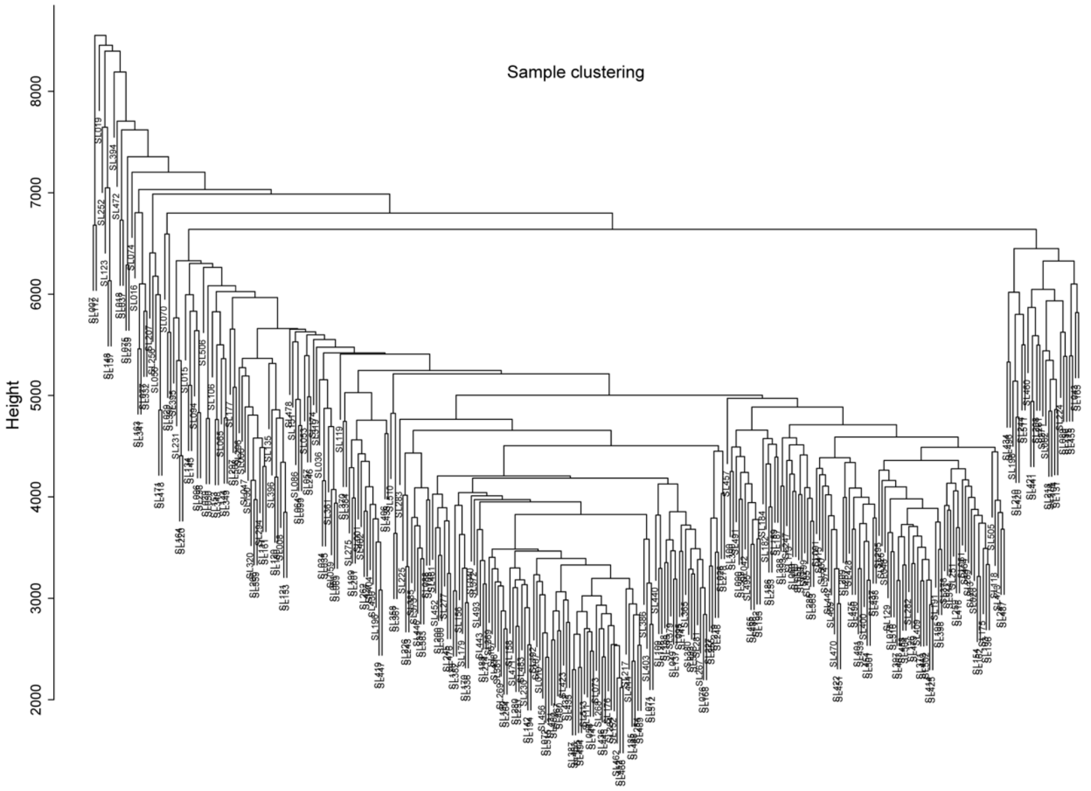

Supplementary Fig. 18 | Genes cluster dendrogram of fruit transcriptome. Gene Cluster dendrogram is based on a dissimilarity measure (1-TOM). The branches represent to modules which is highly interconnected groups of genes.

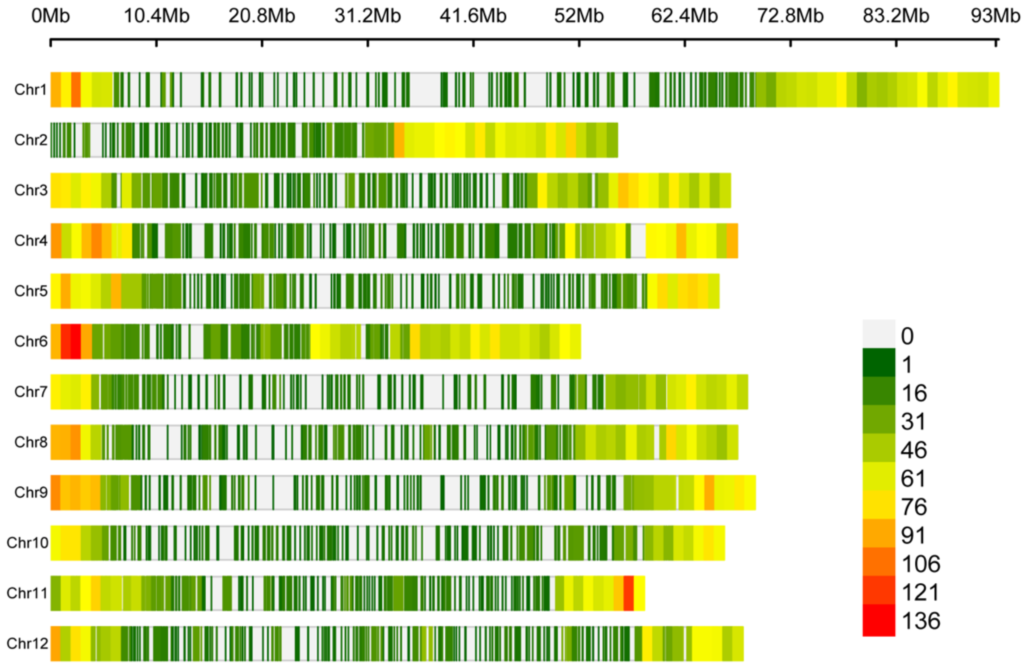

Supplementary Fig. 19 | The distribution plots of candidate SVs for DNA capture array. Colours show the density within 1-Mb window. There are 20,955 candidate SVs comprising 11,488 insertions, 9,403 deletions, and 64 inversions.

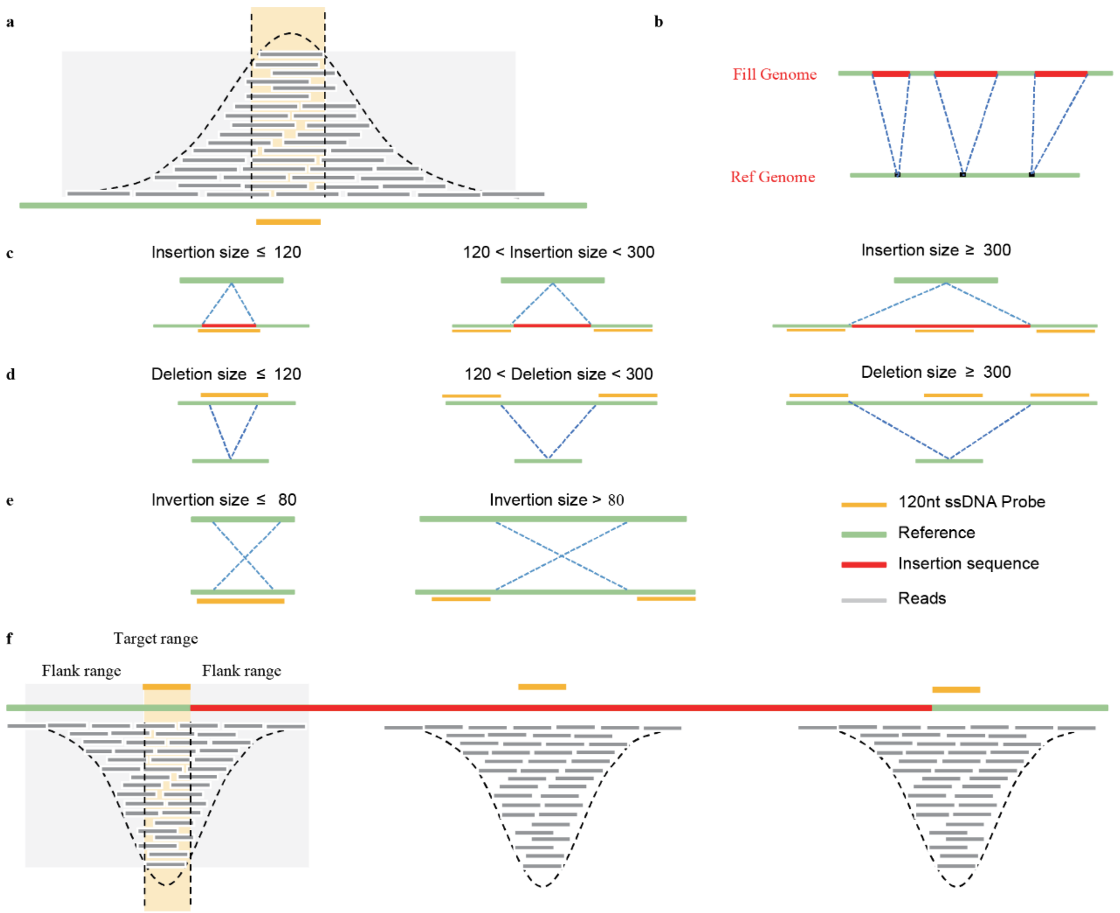

Supplementary Fig. 20 | Conceptual design of DNA capture array. a) Sequencing depth distribution surrounding the area where the probe is designed. The sequencing area is obtained not only for the probe design area (yellow), but also for the flank area of the target area (gray). **b)** Filling of genome. For insertion, we first “fill” the insertion into the reference genome to form a larger pseudo-genome. **c)** Probe design for insertions. For insertions with length shorter than 120-bp, only one probe covering the variation area flanking both sides. For insertions with length ranging from 120-bp to 300-bp, two probes on both flanks of the variation area. For insertions that are longer than 300-bp, three probes are designed. **d)** Probes design for deletions. Probes are designed directly on the reference genome in the same as insertions**. e)** Probes design for inversion. Only one probe covering the area will be designed if the inversion length is shorter than 80-bp. For inversions that are longer than 80-bp, two probes on both flanks of the variation to retrieve inversion information by flank compensation. **f)** The expected reads depth using a filled genome for different types of variants.

Reference

1. Cheng, H., Concepcion, G. T., Feng, X., Zhang, H. & Li, H. Haplotype-resolved de novo assembly using phased assembly graphs with hifiasm. *Nat. Methods* **18**, 170–175 (2021).

2. Nurk, S. *et al.* HiCanu: accurate assembly of segmental duplications, satellites, and allelic variants from high-fidelity long reads. *Genome Res.* **30**, 1291–1305 (2020).

3. Kolmogorov, M., Yuan, J., Lin, Y. & Pevzner, P. A. Assembly of long, error-prone reads using repeat graphs. *Nat. Biotechnol.* **37**, 540–546 (2019).

4. Awad, M. & Gan, X. GALA: gap-free chromosome-scale assembly with long reads. Preprint at https://www.biorxiv.org/content/10.1101/2020.05.15.097428v2 (2020).

5. Guan, D. *et al.* Identifying and removing haplotypic duplication in primary genome assemblies. *Bioinformatics* **36**, 2896–2898 (2020).

6. Chen, S., Zhou, Y., Chen, Y. & Gu, J. fastp: an ultra-fast all-in-one FASTQ preprocessor. *Bioinformatics* **34**, i884–i890 (2018).

7. Durand, N. C. *et al.* Juicebox provides a visualization system for Hi-C contact maps with unlimited zoom. *Cell Syst.* **3**, 99–101 (2016).

8. Yan, Y., Li, Z., Li, Y., Wu, Z. & Yang, R. Correlated evolution of large DNA fragments in the 3D genome of Arabidopsis thaliana. *Mol. Biol. Evol.* **37**, 1621–1636 (2020).

9. Alonge, M. *et al.* RaGOO: fast and accurate reference-guided scaffolding of draft genomes. *Genome Biol.* **20**, 224 (2019).

10. Wang, X. *et al.* Genome of *Solanum pimpinellifolium* provides insights into structural variants during tomato breeding. *Nat. Commun.* **11**, 5817 (2020).

11. Alonge, M. *et al.* Major impacts of widespread structural variation on gene expression and crop improvement in tomato. *Cell* **182**, 145-161 (2020).

12. Ou, S. *et al.* Benchmarking transposable element annotation methods for creation of a streamlined, comprehensive pipeline. *Genome Biol.* **20**, 275 (2019).

13. Ellinghaus, D., Kurtz, S. & Willhoeft, U. LTRharvest, an efficient and flexible software for de novo detection of LTR retrotransposons. *BMC Bioinformatics* **9**, 18 (2008).

14. Ou, S. & Jiang, N. LTR_FINDER_parallel: parallelization of LTR_FINDER enabling rapid identification of long terminal repeat retrotransposons. *Mob. DNA* **10**, 48 (2019).

15. Ou, S. & Jiang, N. LTR_retriever: a highly accurate and sensitive program for identification of long terminal repeat retrotransposons. *Plant Physiol.* **176**, 1410–1422 (2018).

16. Su, W., Gu, X. & Peterson, T. TIR-learner, a new ensemble method for TIR transposable element annotation, provides evidence for abundant new transposable elements in the maize genome. *Mol. Plant* **12**, 447–460 (2019).

17. Xiong, W., He, L., Lai, J., Dooner, H. K. & Du, C. HelitronScanner uncovers a large overlooked cache of Helitron transposons in many plant genomes. *Proc. Natl. Acad. Sci.* **111**, 10263–10268 (2014).

18. Flynn, J. M. *et al.* RepeatModeler2 for automated genomic discovery of transposable element families. *Proc. Natl. Acad. Sci.* **117**, 9451–9457 (2020).

19. Chen, N. Using Repeat Masker to identify repetitive elements in genomic sequences. *Curr. Protoc. Bioinform.* **5**, 4.10.1–4.10.14 (2004).

20. Pease, J. B., Haak, D. C., Hahn, M. W. & Moyle, L. C. Phylogenomics reveals three sources of adaptive variation during a rapid radiation. *PLoS Biol.* **14**, e1002379 (2016).

21. Koenig, D. *et al.* Comparative transcriptomics reveals patterns of selection in domesticated and wild tomato. *Proc. Natl. Acad. Sci.* **110**, E2655–E2662 (2013).

22. Kim, D., Paggi, J. M., Park, C., Bennett, C. & Salzberg, S. L. Graph-based genome alignment and genotyping with HISAT2 and HISAT-genotype. *Nat. Biotechnol.* **37**, 907–915 (2019).

23. Pertea, M. *et al.* StringTie enables improved reconstruction of a transcriptome from RNA-seq reads. *Nat. Biotechnol.* **33**, 290–295 (2015).

24. Niknafs, Y. S., Pandian, B., Iyer, H. K., Chinnaiyan, A. M. & Iyer, M. K. TACO produces robust multisample transcriptome assemblies from RNA-seq. *Nat. Methods* **14**, 68–70 (2017).

25. Hosmani, P. S. *et al.* An improved de novo assembly and annotation of the tomato reference genome using single-molecule sequencing, Hi-C proximity ligation and optical maps. Preprint at

https://www.biorxiv.org/content/10.1101/767764v1.abstract (2019).

26. Pham, G. M. *et al.* Construction of a chromosome-scale long-read reference genome assembly for potato. *Gigascience* **9**, giaa100 (2020).

27. Fu, L., Niu, B., Zhu, Z., Wu, S. & Li, W. CD-HIT: accelerated for clustering the next-generation sequencing data. *Bioinformatics* **28**, 3150–3152 (2012).

28. Holt, C. & Yandell, M. MAKER2: an annotation pipeline and genome-database management tool for second-generation genome projects. *BMC Bioinformatics* **12**, 491 (2011).

29. Hoff, K., Lomsadze, A., Borodovsky, M. & Stanke, M. Whole-genome annotation with BRAKER. *Methods Mol. Biol.* **1962**, 65–95 (2019).

30. Brůna, T., Lomsadze, A. & Borodovsky, M. GeneMark-EP+: eukaryotic gene prediction with self-training in the space of genes and proteins. *NAR genomics Bioinforma.* **2**, lqaa026 (2020).

31. Liu, P., Soukup, A. A., Bresnick, E. H., Dewey, C. N. & Keleş, S. PRAM: a novel pooling approach for discovering intergenic transcripts from large-scale RNA sequencing experiments. *Genome Res.* **30**, 1655–1666 (2020).

32. Zhu, G. *et al.* Rewiring of the fruit metabolome in tomato breeding. *Cell* **172**, 249–261 (2018).

33. Dobin, A. *et al.* STAR: ultrafast universal RNA-seq aligner. *Bioinformatics* **29**, 15–21 (2013).

34. Zhang, R.-G., Wang, Z.-X., Ou, S. & Li, G.-Y. TEsorter: lineage-level classification of transposable elements using conserved protein domains. Preprint at https://www.biorxiv.org/content/10.1101/800177v1.abstract (2019).

35. Haas, B. J. *et al.* De novo transcript sequence reconstruction from RNA-seq using the Trinity platform for reference generation and analysis. *Nat. Protoc.* **8**, 1494–1512 (2013).

36. UniProt: the universal protein knowledgebase in 2021. *Nucleic Acids Res.* **49**, D480–D489 (2021).

37. Buchfink, B., Xie, C. & Huson, D. H. Fast and sensitive protein alignment using DIAMOND. *Nat. Methods* **12**, 59–60 (2015).

38. Jones, P. *et al.* InterProScan 5: genome-scale protein function classification. *Bioinformatics* **30**, 1236–1240 (2014).

39. Emms, D. M. & Kelly, S. OrthoFinder: phylogenetic orthology inference for comparative genomics. *Genome Biol.* **20**, 238 (2019).

40. Rhie, A., Walenz, B. P., Koren, S. & Phillippy, A. M. Merqury: reference-free quality, completeness, and phasing assessment for genome assemblies. *Genome Biol.* **21**, 245 (2020).

41. Waterhouse, R. M. *et al.* BUSCO applications from quality assessments to gene prediction and phylogenomics. *Mol. Biol. Evol.* **35**, 543–548 (2018).

42. Mapleson, D., Garcia Accinelli, G., Kettleborough, G., Wright, J. & Clavijo, B. J. KAT: a K-mer analysis toolkit to quality control NGS datasets and genome assemblies. *Bioinformatics* **33**, 574–576 (2017).

43. Li, H. Minimap2: pairwise alignment for nucleotide sequences. *Bioinformatics* **34**, 3094–3100 (2018).

44. Poplin, R. *et al.* A universal SNP and small-indel variant caller using deep neural networks. *Nat. Biotechnol.* **36**, 983–987 (2018).

45. Purcell, S. *et al.* PLINK: a tool set for whole-genome association and population-based linkage analyses. *Am. J. Hum. Genet.* **81**, 559–575 (2007).

46. Narasimhan, V. *et al.* BCFtools/RoH: a hidden Markov model approach for detecting autozygosity from next-generation sequencing data. *Bioinformatics* **32**, 1749–1751 (2016).

47. Garrison, E. *et al.* Variation graph toolkit improves read mapping by representing genetic variation in the reference. *Nat. Biotechnol.* **36**, 875–879 (2018).

48. Sirén, J. *et al.* Pangenomics enables genotyping of known structural variants in 5202 diverse genomes. *Science* **374**, eabg8871 (2021).

49. Yue, J.-X. & Liti, G. simuG: a general-purpose genome simulator. *Bioinformatics* **35**, 4442–4444 (2019).

50. Huang, W., Li, L., Myers, J. R. & Marth, G. T. ART: a next-generation sequencing read simulator. *Bioinformatics* **28**, 593–594 (2012).

51. Zook, J. M. *et al.* A robust benchmark for detection of germline large deletions and insertions. *Nat. Biotechnol.* **38**, 1347–1355 (2020).

52. Sim, S.-C. *et al.* Development of a large SNP genotyping array and generation of high-density genetic maps in tomato. *PLoS One* **7**, e40563 (2012).

53. Speed, D., Hemani, G., Johnson, M. R. & Balding, D. J. Improved heritability estimation from genome-wide SNPs. *Am. J. Hum. Genet.* **91**, 1011–1021 (2012).

54. Bastian, M., Heymann, S. & Jacomy, M. Gephi: an open source software for exploring and manipulating networks. in *Third international AAAI conference on weblogs and social media* (2009).

55. Stamatakis, A. RAxML version 8: a tool for phylogenetic analysis and post-analysis of large phylogenies. *Bioinformatics* **30**, 1312–1313 (2014).
