# Supplementary material for: Graph pangenome captures missing heritability and empowers tomato breeding
Source: Nature. 2022 Jun 8;606(7914):527–34. doi: 10.1038/s41586-022-04808-9 (PMC9200638; doi:10.1038/s41586-022-04808-9)
Supplement: Supplementary file 2 — Reporting Summary [file 41586_2022_4808_MOESM2_ESM.pdf]

nature portfolio

Corresponding author(s): Sanwen Huang

Last updated by author(s): Feb 28, 2022

Reporting Summary

Nature Portfolio wishes to improve the reproducibility of the work that we publish. This form provides structure for consistency and transparency in reporting. For further information on Nature Portfolio policies, see our Editorial Policies and the Editorial Policy Checklist.

Statistics

For all statistical analyses, confirm that the following items are present in the figure legend, table legend, main text, or Methods section.

n/a	Confirmed	
☐	☒	The exact sample size (*n*) for each experimental group/condition, given as a discrete number and unit of measurement
☒	☐	A statement on whether measurements were taken from distinct samples or whether the same sample was measured repeatedly
☐	☒	The statistical test(s) used AND whether they are one- or two-sided<br>*Only common tests should be described solely by name; describe more complex techniques in the Methods section.*
☐	☒	A description of all covariates tested
☐	☒	A description of any assumptions or corrections, such as tests of normality and adjustment for multiple comparisons
☐	☒	A full description of the statistical parameters including central tendency (e.g. means) or other basic estimates (e.g. regression coefficient) AND variation (e.g. standard deviation) or associated estimates of uncertainty (e.g. confidence intervals)
☐	☒	For null hypothesis testing, the test statistic (e.g. *F*, *t*, *r*) with confidence intervals, effect sizes, degrees of freedom and *P* value noted<br>*Give P values as exact values whenever suitable.*
☐	☒	For Bayesian analysis, information on the choice of priors and Markov chain Monte Carlo settings
☒	☐	For hierarchical and complex designs, identification of the appropriate level for tests and full reporting of outcomes
☒	☐	Estimates of effect sizes (e.g. Cohen's *d*, Pearson's *r*), indicating how they were calculated

Our web collection on statistics for biologists contains articles on many of the points above.

Software and code

Policy information about availability of computer code

Data collection	32 tomato accessions were sequenced with High-fidelity (HiFi) long reads.
Data analysis	minimap2 (v2.17-r941), LDAK (v5.1), GCTA (v1.93.2), WGCNA (v1.70-3), GALA (v1.0.0), MAKER2 (v3.01.03), PRAM, LiftOver, Paragraph(v2.2b), minigraph (v0.14-r415), rrBLUP(v4.6.1), Flye (v2.7), Hicanu (v2.0), Hifiasm (v0.13), RagTag (v1.0.1), HISAT2 (v2.10.2), StringTie (v1.3.0), TACO (v0.7.3), SNAP (v2006-07-28), Augustus (v3.3.3), CD-HIT (v4.6), BRAKER (v2.1.3), GeneMark-ET/ES (v4.3.8), DeepVariant (v0.9.0, v1.0.0), NGLMR (v0.2.7), Sniffles (v1.0.12), SVIM (v1.2.0), CuteSV (v1.0.10), PBSV(v2.4.0), SURVIVOR (v1.0.6), Mash (v2.2), Kallisto (v0.46.2), Plink (v2.0), ccs (v6.0.0), MUMmer (v4.0). The custom code is available at https://github.com/YaoZhou89/TGG.

For manuscripts utilizing custom algorithms or software that are central to the research but not yet described in published literature, software must be made available to editors and reviewers. We strongly encourage code deposition in a community repository (e.g. GitHub). See the Nature Portfolio guidelines for submitting code & software for further information.

Data

Policy information about availability of data

All manuscripts must include a data availability statement. This statement should provide the following information, where applicable:

- Accession codes, unique identifiers, or web links for publicly available datasets
- A description of any restrictions on data availability
- For clinical datasets or third party data, please ensure that the statement adheres to our policy

All sequence data generated in this study have been deposited at the Sequence Read Archive (https://ncbi.nlm.nih.gov/sra) under BioProject PRJNA733299. Whole-genome sequencing data was downloaded from NCBI (BioProjects PRJNA259308, PRJNA353161, PRJNA454805 and PRJEB5235) and RNA-seq data was downloaded from the NCBI (BioProject PRJNA396272). All assemblies with annotations, variants VCF files and graph files are available at SolOmics database (http://

solomics.agis.org.cn/tomato/ftp) and Sol Genomics Network (https://solgenomics.net/ftp/genomes/TGG/). InterPro database is downloaded from https://www.ebi.ac.uk/interpro/. The UniProtKB/SwissProt database is downloaded from https://www.uniprot.org.

Field-specific reporting

Please select the one below that is the best fit for your research. If you are not sure, read the appropriate sections before making your selection.

☒ Life sciences ☐ Behavioural & social sciences ☐ Ecological, evolutionary & environmental sciences

For a reference copy of the document with all sections, see nature.com/documents/nr-reporting-summary-flat.pdf

Life sciences study design

All studies must disclose on these points even when the disclosure is negative.

Sample size	No statistical methods were used to establish sample size. The 32 accessions used for assembly were selected to represent the genetic diversity of tomato. We downloaded the public available resequencing data, RNA-seq data, and metabolomics data and accessions with all three types of data were used for association and heritability analysis.
Data exclusions	No data were excluded.
Replication	We confirmed the ability to replicate all code of this study.
Randomization	Randomization does not directly apply to the genome sequencing and assembly.
Blinding	No analyses required being blind to groups.

Reporting for specific materials, systems and methods

We require information from authors about some types of materials, experimental systems and methods used in many studies. Here, indicate whether each material, system or method listed is relevant to your study. If you are not sure if a list item applies to your research, read the appropriate section before selecting a response.

Materials & experimental systems

n/a	Involved in the study	
☒	☐	Antibodies
☒	☐	Eukaryotic cell lines
☒	☐	Palaeontology and archaeology
☒	☐	Animals and other organisms
☒	☐	Human research participants
☒	☐	Clinical data
☒	☐	Dual use research of concern

Methods

n/a	Involved in the study	
☒	☐	ChIP-seq
☒	☐	Flow cytometry
☒	☐	MRI-based neuroimaging

March 2021
